# Epitranscriptomic m^5^C methylation of SARS-CoV-2 RNA regulates viral replication and the virulence of progeny viruses in the new infection

**DOI:** 10.1126/sciadv.adn9519

**Published:** 2024-08-07

**Authors:** Hongyun Wang, Jiangpeng Feng, Zhiying Fu, Tianmo Xu, Jiejie Liu, Shimin Yang, Yingjian Li, Jikai Deng, Yuzhen Zhang, Ming Guo, Xin Wang, Zhen Zhang, Zhixiang Huang, Ke Lan, Li Zhou, Yu Chen

**Affiliations:** ^1^ State Key Laboratory of Virology, Modern Virology Research Center and RNA Institute, College of Life Sciences and Frontier Science Center for Immunology and Metabolism, Wuhan University, Wuhan 430072, China.; ^2^ Institute for Vaccine Research, Animal Bio-Safety Level III Laboratory at Center for Animal Experiment, Wuhan University, Wuhan 430071, China.

## Abstract

While the significance of N6-methyladenosine (m^6^A) in viral regulation has been extensively studied, the functions of 5-methylcytosine (m^5^C) modification in viral biology remain largely unexplored. In this study, we demonstrate that m^5^C is more abundant than m^6^A in severe acute respiratory syndrome coronavirus 2 (SARS-CoV-2) and provide a comprehensive profile of the m^5^C landscape of SARS-CoV-2 RNA. Knockout of NSUN2 reduces m^5^C levels in SARS-CoV-2 virion RNA and enhances viral replication. *Nsun2* deficiency mice exhibited higher viral burden and more severe lung tissue damages. Combined RNA-Bis-seq and m^5^C-MeRIP-seq identified the NSUN2-dependent m^5^C-methylated cytosines across the positive-sense genomic RNA of SARS-CoV-2, and the mutations of these cytosines enhance RNA stability. The progeny SARS-CoV-2 virions from *Nsun2* deficiency mice with low levels of m^5^C modification exhibited a stronger replication ability. Overall, our findings uncover the vital role played by NSUN2-mediated m^5^C modification during SARS-CoV-2 replication and propose a host antiviral strategy via epitranscriptomic addition of m^5^C methylation to SARS-CoV-2 RNA.

## Teaser

The host m^5^C methyltransferase NSUN2 regulates m^5^C modification, viral replication, and pathogenesis of SARS-CoV-2.

## INTRODUCTION

COVID-19 caused by severe acute respiratory syndrome coronavirus 2 (SARS-CoV-2) has become a globe health challenge since December 2019 and is still going on (*1*–*3*). SARS-CoV-2 belongs to betacoronaviruses, containing a single-stranded positive-sense genomic RNA (gRNA) of approximately 30 kb with 5′-cap structure and 3′-poly-A tail (*4*). The positive-sense gRNA is used as the template to translate polyprotein 1a/1ab (pp1a/pp1ab) (*5*). The two large open reading frames (ORF1a/1b) near the 5′ terminus of gRNA encode pp1a/pp1ab, which is then cleaved into 16 viral nonstructural proteins (nsp1 to nsp16) by nsp3 harboring papain-like protease (PLP^pro^) activity and nsp5 harboring 3C-like protease (3CL^pro^) activity (*6*). The nsps form the replication-transcription complex. Subsequently, the subgenomic RNAs (sgRNAs) are generated by ORFs the near the 3′ terminus of gRNA through transcription-regulating sequence (TRS)–dependent template switching by the replication-transcription complex and encode four main structural proteins, including spike (S), envelope (E), membrane (M), nucleocapsid (N), and several accessory proteins (*7*). The positive-sense gRNA also serves as the template for replication and transcription to produce the negative-sense RNA intermediates by nsp12 harboring RNA-dependent RNA polymerase activity. The negative-sense RNA intermediates subsequently serve as the templates to produce positive-sense gRNA and sgRNAs (*8*).

More than 170 types of RNA chemical modifications to various types of RNAs have been characterized thus far, which are involved in physiological and pathological processes via regulating RNA metabolism, including stability, translation efficiency, alternative splicing, transport, and localization (*9*–*12*). N6-methyladenosine (m^6^A) and 5-methylcytosine (m^5^C) are particularly notable and extensively existed (*13*, *14*). m^6^A has been reported to be involved in the modification and regulation of a large variety of viruses, including HIV (*Retroviridae*) (*15*–*19*), hepatitis B virus (HBV, *Hepadnaviridae*) (*20*), Zika virus (*Flaviviridae*) (*21*, *22*), human metapneumovirus (*Pneumoviridae*) (*23*), Kaposi’s sarcoma-associated herpesvirus (*Herpesviridae*) (*24*–*26*), enterovirus 71 (*Picornaviridae*) (*27*), influenza A virus (*Orthomyxoviridae*) (*28*, *29*), human respiratory syncytial virus (*Paramyxoviridae*) (*30*, *31*), endogenous retroviruses (*Retroviridae*) (*32*), polyomavirus simian virus 40 (*Polyomaviridae*) (*33*), and so on. Especially recently, m^6^A were reported to be widely distributed in SARS-CoV-2 gRNA. Although inconsistent results of the METTL3-mediated regulation to the replication of SARS-CoV-2 have been demonstrated, all these studies indicate that m^6^A modification plays an important regulatory function in SARS-CoV-2 life cycle (*34*–*40*). However, compared to m^6^A, little is known about the functions of m^5^C modification machinery in most physiological and pathological contexts upon various viral infections. So far, only a few viruses, including HIV (*41*, *42*), HBV (*43*), murine leukemia virus (*Retroviridae*) (*44*, *45*), and Epstein-Barr virus (*Herpesviridae*) (*46*), have been reported to be modified and regulated by m^5^C modification machinery. However, whether there is m^5^C methylation modification in SARS-CoV-2 gRNA or the m^5^C modification participates in the regulation of SARS-CoV-2 replication and pathogenic processes remains to be investigated.

In this study, we characterized the m^5^C RNA modifications present in SARS-CoV-2 using mass spectrometry (MS) and next-generation sequencing. Our findings revealed that NSUN2, a typical RNA m^5^C methyltransferase, functions as a negative regulator of SARS-CoV-2 replication. We profiled the widespread distribution of m^5^C methylation sites in positive-sense SARS-CoV-2 gRNA. Mechanistically, NSUN2 mediated the methylation of SARS-CoV-2 RNA transcripts and facilitated their degradation. Knockout of NSUN2 notably reduced m^5^C levels in SARS-CoV-2 virion RNA and increased viral infection. By using the transcription and replication-competent SARS-CoV-2 virus–like particles (trVLP) system, we found that the SARS-CoV-2 green fluorescent protein (GFP)/ΔN trVLPs with low NSUN2-mediated m^5^C modification exhibited higher replication ability. Consistently, the progeny SARS-CoV-2 virions from *Nsun2^+/−^* mice (with low m^5^C level) exhibited a stronger replication ability in both Caco-2 cells and K18-hACE2 mice than the progeny SARS-CoV-2 virions from *Nsun2^+/+^* mice (with normal m^5^C level). Overall, our findings highlight the vital role of NSUN2-mediated m^5^C methylation during SARS-CoV-2 replication and pathogenesis. We outline a paradigm that host has evolved an antiviral strategy via epitranscriptomic addition of m^5^C methylation to SARS-CoV-2 RNA, which will be an insight valuable for developing new anti-coronavirus drugs.

## RESULTS

### SARS-CoV-2 gRNA contains more abundant m^5^C modification compared to m^6^A and other modifications

**Fig. 1.**
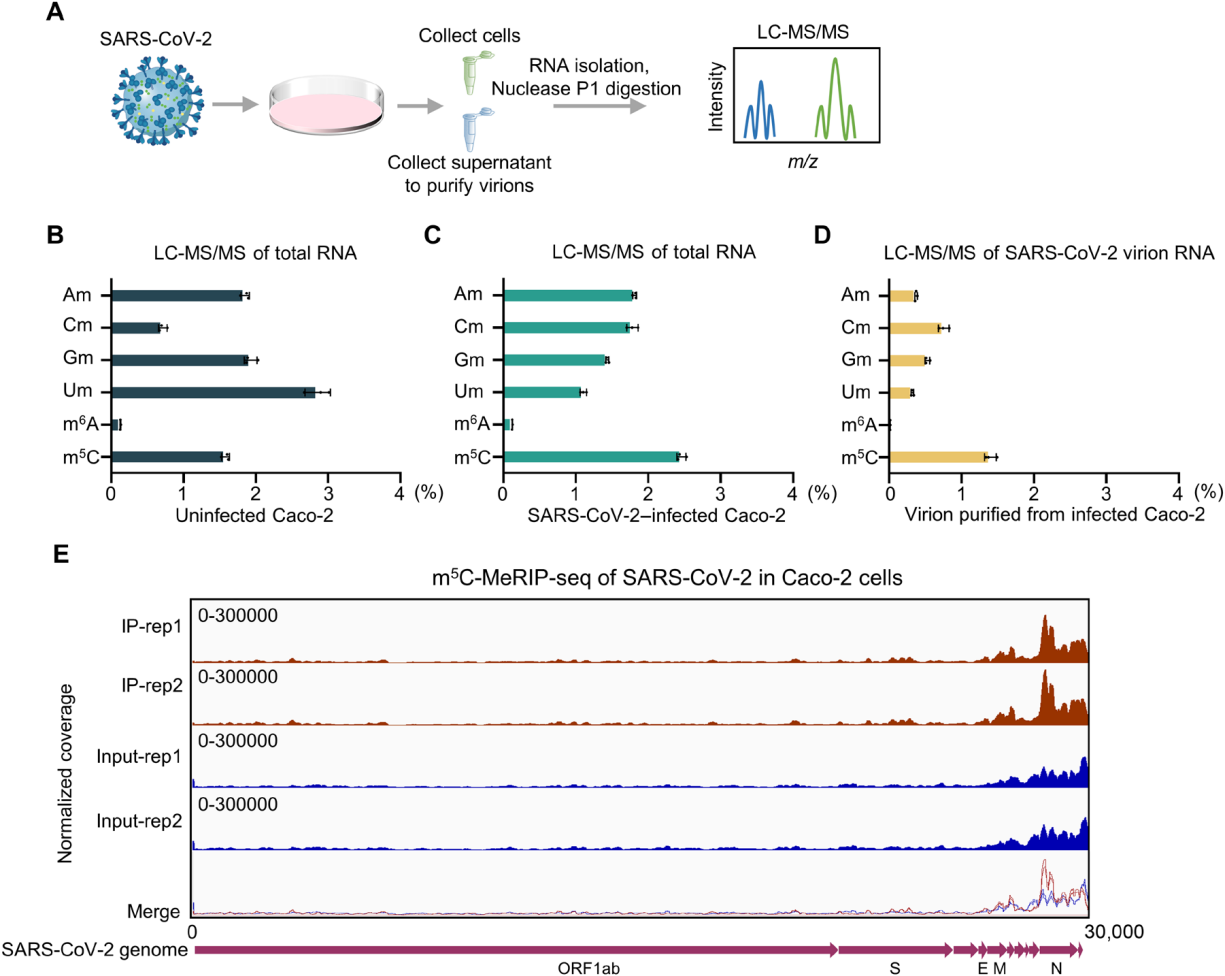
SARS-CoV-2 gRNA contains abundant m^5^C modification. (**A**) Schematic diagram of LC-MS/MS of purified SARS-CoV-2 virions or SARS-CoV-2–infected Caco-2 cells at multiplicity of infection (MOI) = 0.02 for 48 hour. (**B**) Analysis of m^5^C/C ratio using LC-MS/MS of total RNAs extracted from uninfected Caco-2 cells. (**C** and **D**) Analysis of m^5^C/C ratio using LC-MS/MS of total RNAs extracted from SARS-CoV-2–infected Caco-2 cells (C) or purified SARS-CoV-2 virion RNA (D). (**E**) Visualization of m^5^C-MeRIP-seq results shows regions of enrichment for m^5^C immunoprecipitation (upper) over input (lower) from SARS-CoV-2–infected Caco-2 cells. Integrative Genomics Viewer tracks displaying read distributions from m^5^C-MeRIP-seq (red panel) and Input (blue panel) along SARS-CoV-2 positive-sense RNA. Data are representative of three independent experiments and were analyzed by two-tailed unpaired *t* test. Graphs show the means ± SD (*n* = 3) derived from three independent experiments [or two independent experiments for (E)].

To investigate RNA modifications that SARS-CoV-2 RNA contains, we performed liquid chromatography tandem MS (LC-MS/MS) of SARS-CoV-2–infected Caco-2 cells or purified virions (Fig. 1A). We identified several modified ribonucleosides in SARS-CoV-2 RNA, including 2′-O–methylated derivatives of four canonical nucleosides (Am, Cm, Um, and Gm), m^5^C, and m^6^A. Compared to total RNA of uninfected Caco-2 cells (Fig. 1B), m^5^C is the most abundant RNA modification both in total RNA of SARS-CoV-2–infected Caco-2 cells (Fig. 1C) or purified SARS-CoV-2 virion RNA (Fig. 1D) among these modifications. Next, we developed a biosafety level 2 (BSL-2) cell culture system for the production of trVLP, which expresses GFP protein replacing viral N protein (SARS-CoV-2 GFP/ΔN trVLP). We also purified SARS-CoV-2 GFP/ΔN trVLP virion and identified many modified ribonucleosides in its RNA. Consistently, m^5^C is the most abundant modification compared with many other RNA modifications (fig. S1). Furthermore, we performed m^5^C-methylated RNA immunoprecipitation sequencing (m^5^C-MeRIP-seq) in Caco-2 cells, which were infected with SARS-CoV-2 for 48 hours to profile the m^5^C landscape along SARS-CoV-2 transcriptome. As shown in Fig. 1E, the m^5^C is enriched in the region near the 3′ untranslated region.

### NSUN2 inhibited SARS-CoV-2 replication and regulated m^5^C modification of SARS-CoV-2 RNA

**Fig. 2.**
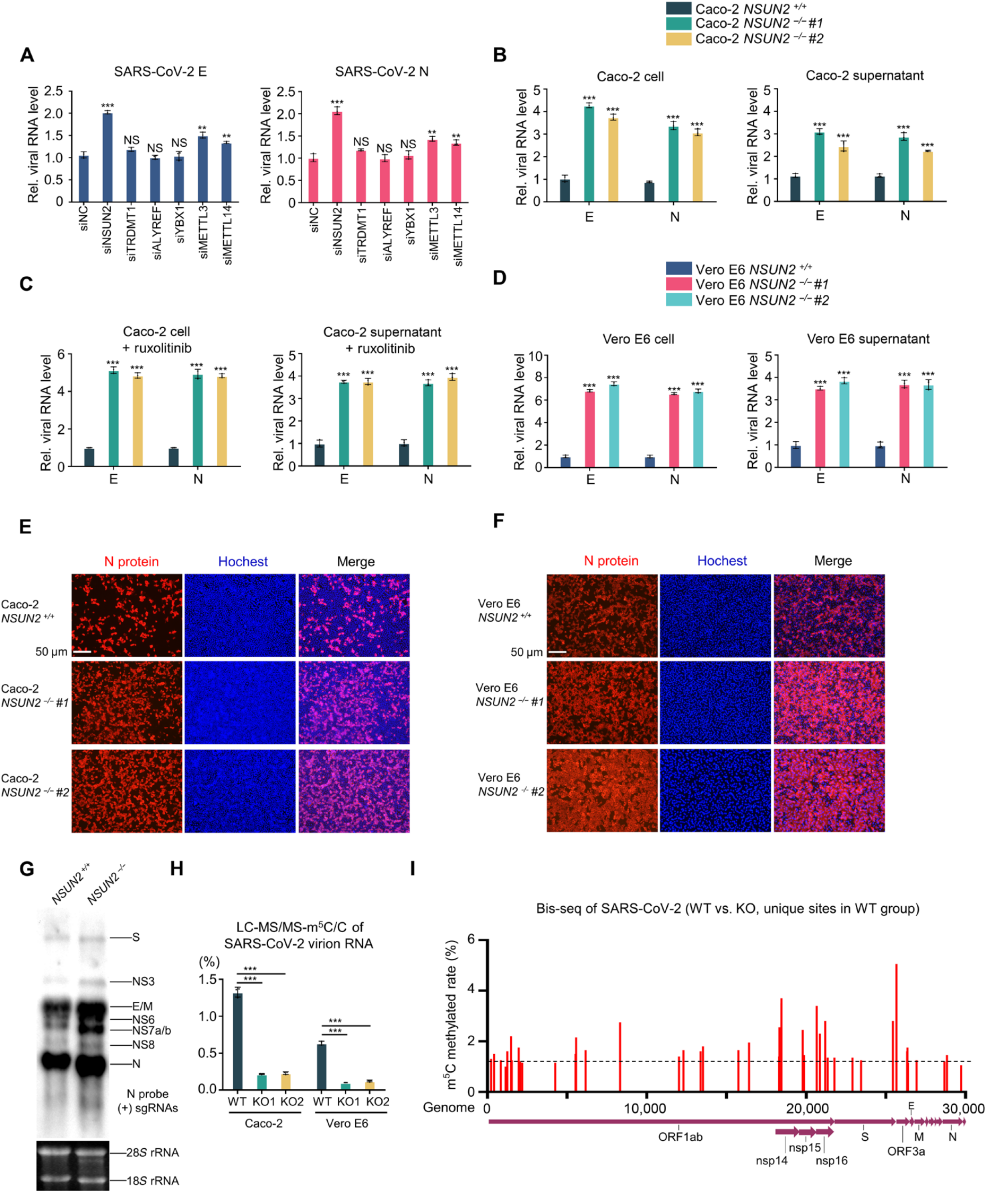
NSUN2 regulated m^5^C modification and replication of SARS-CoV-2. (**A**) qPCR analysis of RNA levels of N or E in Caco-2 cells transfected with siControl or siRNAs for 36 hours, with infection by SARS-CoV-2 at an MOI = 0.02 for another 24 hours. (**B** and **C**) qPCR analysis of RNA levels of N or E in cell or supernatant from WT Caco-2 cells or NSUN2 knockout cells, with infection by SARS-CoV-2 at an MOI = 0.02 for 24 hours, with (B) or without (C) ruxolitinib treatment. (**D**) qPCR analysis of RNA levels of N or E in cell or supernatant from WT Vero E6 cells or NSUN2 knockout cells, with infection by SARS-CoV-2 at an MOI = 0.02 for 24 hours. (**E**) Immunofluorescence analysis of N protein (red) in WT Caco-2 cells or NSUN2 knockout cells, with infection by SARS-CoV-2 at an MOI = 0.02 for 24 hours. Scale bar, 50 μm. (**F**) Immunofluorescence analysis of N protein (red) in WT Vero E6 cells or NSUN2 knockout cells, with infection by SARS-CoV-2. Scale bar, 50 μm. (**G**) Northern blot results from RNA probes targeting (+) sgRNAs (N probe) in WT Caco-2 cells or NSUN2 knockout cells, with infection by SARS-CoV-2. (**H**) Analysis of m^5^C/C ratio using LC-MS/MS of purified SARS-CoV-2 virion RNA from WT cells or NSUN2 knockout cells. (**I**) Bis-seq results of unique m^5^C-methylated sites along SARS-CoV-2 positive-sense RNA in SARS-CoV-2–infected WT Caco-2 cells versus NSUN2 knockout Caco-2 cells. Data are representative of three independent experiments and were analyzed by two-tailed unpaired *t* test. Graphs show the means ± SD (*n* = 3) derived from three independent experiments [or two independent experiments for (I)]. NS, not significant for *P* > 0.05, **P < 0.01, ***P < 0.001.

To further investigate whether m^5^C machinery regulates SARS-CoV-2 infection and replication, we knocked down currently known writers or readers (*47*, *48*) of mRNA m^5^C modification and NOP2/SUN RNA methyltransferase family members (*49*) in Caco-2 cells using small interfering RNAs (siRNAs) and detected the endogenous viral RNA levels of SARS-CoV-2. Knockdown of NSUN2 enhanced endogenous SARS-CoV-2 N and E RNA levels, while the other currently known writers or readers did not (Fig. 2A and fig. S2). Notably, knockdown of METTL3 or METTL14 only mildly enhanced endogenous SARS-CoV-2 N and E RNA levels. We thus speculated that NSUN2 may specifically and notably regulate SARS-CoV-2 replication compared to other currently known methyltransferases or readers. We then constructed NSUN2 knockout cell lines in Caco-2 cells using CRISPR-Cas9 (fig. S3) and found that knockout of NSUN2 showed notable elevation of the RNA levels of SARS-CoV-2 N and E intracellularly and extracellularly (Fig. 2B). Consistently, knockout of NSUN2 dramatically elevated the protein levels of SARS-CoV-2 N (Fig. 2E).

Our previous study identified that NSUN2 participates in the regulation of virus-induced innate immunity responses (*50*). To block out the effects of interferon, we used ruxolitinib, a potent and selective Janus kinase 1/2 inhibitor that blocks signaling downstream of type I interferon receptors. As shown in Fig. 2C, the promotion of SARS-CoV-2 propagation in NSUN2-knockout cells was not affected by ruxolitinib treatment. This result further confirmed that promotion of SARS-CoV-2 propagation in the NSUN2-deficient cells was independent of the interferon response. Moreover, we also constructed NSUN2 knockout cell lines in Vero E6 cell, which is deficient in interferon secretion, and the same results were also obtained in NSUN2-knockout Vero E6 cell lines compared with wild-type (WT) Vero E6 cells (Fig. 2, D and F). Meanwhile, we observed that the promotion of SARS-CoV-2 propagation in NSUN2-knockout cells was mildly increased when blocking out the effects of interferon, further demonstrating the notable direct NSUN2-mediated regulation of SARS-CoV-2 replication (Fig. 2, B to D). In addition, to explore the global landscape change of sgRNAs after NSUN2 knockout, we verified the presence of sgRNAs in SARS-CoV-2–infected Caco-2 cells using Northern blotting. The result showed that the global landscape of sgRNAs in NSUN2-knockout cells notably increased compared to WT Caco-2 cells (Fig. 2G). The replication of SARS-CoV-2 takes place at endoplasmic reticulum–derived double-membrane vesicles (ER-DMVs). We further checked whether NSUN2 exists in ER-DMVs. We purified the ER-DMVs from SARS-CoV-2–infected cells, and NSUN2 protein was identified in the ER-DMVs (fig. S4A). Moreover, we also perform nuclear and cytoplasmic protein extraction to investigate the subcellular localization of endogenous NSUN2 upon SARS-CoV-2 infection. Consistent with previous results, NSUN2 were detected mostly in the nucleus in uninfected cells. However, upon SARS-CoV-2 infection, the distribution of NSUN2 has changed and is more distributed in the cytoplasm (fig. S4B). These results further demonstrate the colocalization and critical role of NSUN2 in regulation of SARS-CoV-2 replication.

To assess the m^5^C level of SARS-CoV-2 gRNA in virion from WT or NSUN2 knockout cell lines, we further purified SARS-CoV-2 virions released from Vero E6 cell line and Caco-2 cell line and corresponding NSUN2 knockout cell lines. The LC-MS/MS results provided direct evidence of a notable decrease in m^5^C levels in virion RNA after NSUN2 knockout (Fig. 2H). To further confirm the specific m^5^C modification sites, we performed RNA-bisulfite sequencing (RNA-Bis-seq) both in WT Caco-2 cells and NSUN2 knockout Caco-2 cells that were infected with SARS-CoV-2 (Fig. 2I and figs. S5 and S6). We identified a large amount of m^5^C-methylated sites among SARS-CoV-2 gRNA. Here, we showed the unique m^5^C-methylated sites of positive-sense SARS-CoV-2 gRNA in WT groups versus NSUN2 knockout groups (Fig. 2I). These sites were mediated by NSUN2 and scattered across positive-sense gRNA. The relatively high methylation sites were mainly distributed in nsp14, nsp15, nsp16, S, ORF3a, E, M, and N.

### The screened m^5^C-methylated cytosines in SARS-CoV-2 were identified to regulate RNA degradation

**Fig. 3.**
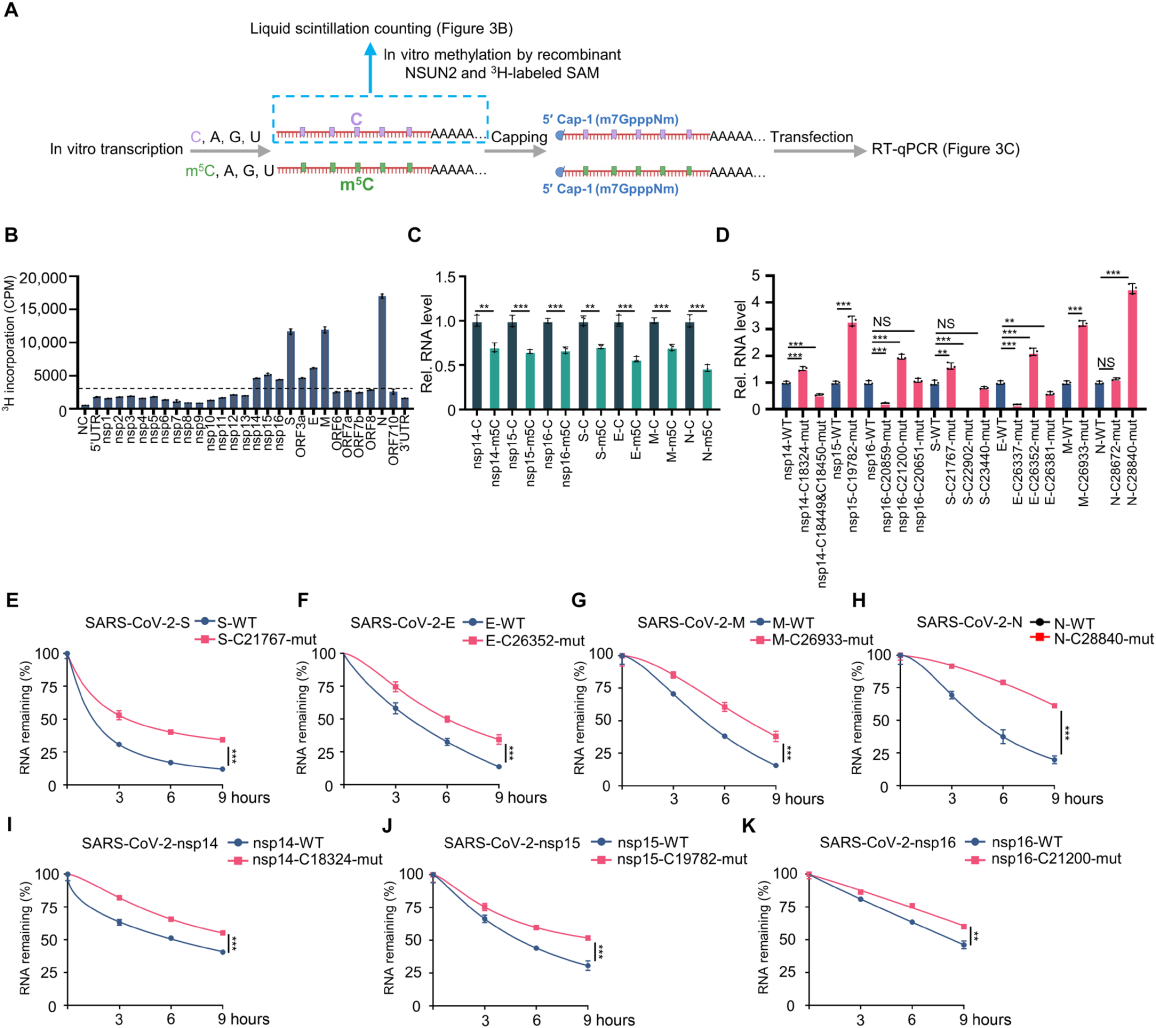
NSUN2 regulated SARS-CoV-2 RNA degradation. (**A**) Schematic diagram of in vitro–transcribed RNAs with C or m^5^C using in vitro methylation assays or transfection. (**B**) In vitro m^5^C methylation assays using recombinant GST-NSUN2 and the in vitro transcripts as indicated. (**C**) qPCR analysis of RNA levels of the corresponding gene in Huh7 cells transfected with transcribed RNAs with C or m^5^C for 10 hours. (**D**) qPCR analysis of RNA levels of the corresponding gene in Huh7 cells transfected with WT or m^5^C modification site-mutations (mut) of each gene. (**E** to **K**) Stability analysis of RNA of each gene in Huh7 cells transfected with WT or m^5^C modification site-mutations (mut) of each gene for 24 hours, with treatment of actinomycin D (ActD) for another 0, 3, 6, and 9 hours. Data are representative of three independent experiments and were analyzed by two-tailed unpaired *t* test. Graphs show the means ± SD (*n* = 3) derived from three independent experiments. NS, not significant for *P* > 0.05, **P < 0.01, ***P < 0.001.

We then sought to study the function of these relatively high m^5^C methylation sites. We prepared RNA segments of each region of SARS-CoV-2 gRNA by in vitro transcription. The RNAs were used for in vitro methylation assays using recombinant glutathione *S*-transferase (GST)–NSUN2 and ^3^H-labeled *S*-adenosyl methionine (SAM). These transcribed RNA segments can be methylated by NSUN2 (Fig. 3, A and B). Meanwhile, the data showed that NSUN2 can more efficiently mediate the methylation of N in vitro compared with other regions. Furthermore, we prepared RNA segments with C or m^5^C. These transcribed RNA segments with C or m^5^C were transfected into cells after capping (Fig. 3, A and C, and figs. S7 and S8). The quantitative polymerase chain reaction (qPCR) results in the expression levels of these transcribed RNA segments with m^5^C in nsp14, nsp15, nsp16, S, E, M, and N being lower than those corresponding transcribed RNA segments with C (Fig. 3C and figs. S7 and S8). However, the incorporation of m^5^C mildly promoted the RNA stability of ORF3a but did not affect the RNA degradation of nsp6 (with no notable NSUN2-dependent m^5^C site), indicating that not all m^5^C cause degradation (fig. S7). We speculated that incorporation of m^5^C into these transcribed RNA segments may affect the stability of RNA of nsp14, nsp15, nsp16, S, E, M, and N. We then tested whether these identified highly methylated cytosines were indeed involved in the regulation of mRNA stability. We constructed expression plasmids containing either WT or site mutations (table S2) of methylated cytosines of nsp14, nsp15, nsp16, S, E, M, or N and found that most of the site mutations of methylated cytosines increased the corresponding RNA expression levels (Fig. 3D). We then measured the stability of these transcripts and found that the transcripts of site mutation of methylated cytosines were consistently more stable than the WT transcripts, indicating that methylation of these cytosines by NSUN2 was indeed critical for regulating their mRNA stability and degradation (Fig. 3, E to K, and fig. S9).

**Fig. 4.**
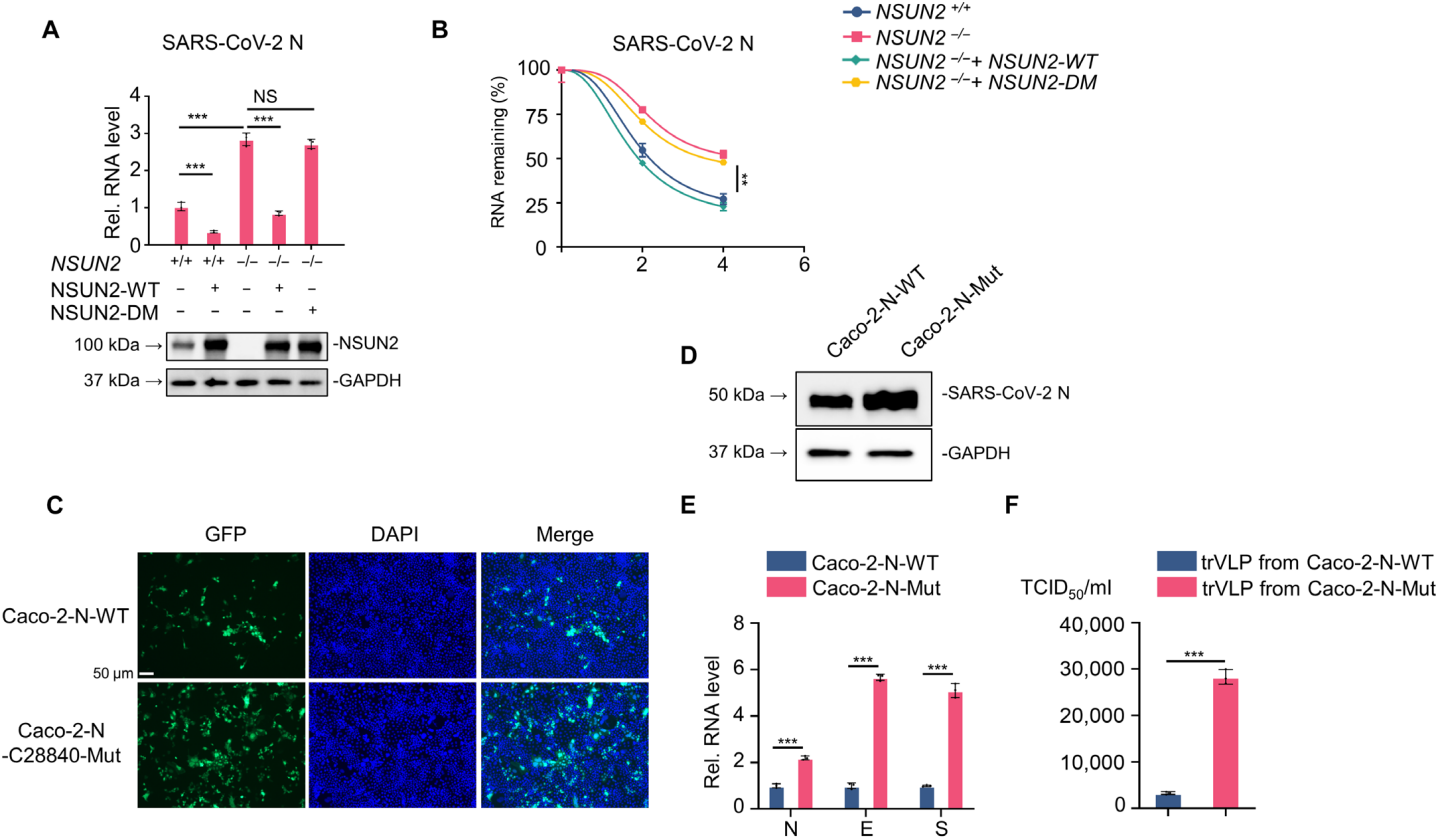
NSUN2 regulated RNA level of SARS-CoV-2 N gene depending on its methyltransferase activity. (**A**) qPCR analysis of RNA levels of N gene in WT Vero E6 cells (*NSUN2^+/+^*) or NSUN2 knockout Vero E6 cells (*NSUN2^−/−^*), transfected with SARS-CoV-2 N and WT NSUN2 (NSUN2-WT) or I302A/C321A double mutation (NSUN2-DM) for 36 hours. (**B**) Stability analysis of RNA levels of N gene in WT Vero E6 cells (*NSUN2^+/+^*) or NSUN2 knockout Vero E6 cells (*NSUN2^−/−^*), transfected with SARS-CoV-2 N and WT NSUN2 (NSUN2-WT) or I302A/C321A double mutation (NSUN2-DM) for 24 hours, with treatment of actinomycin D (ActD) for another 0, 2, and 4 hours. (**C**) Immunofluorescence analysis of SARS-CoV-2 GFP/ΔN trVLP (green) in Caco-2 cells reconstituted with WT N (N-WT) or N-C28840-Mut by lentiviral system, with infection by SARS-CoV-2 GFP/ΔN trVLP at an MOI = 0.05 for 48 to 72 hours. Scale bar, 50 μm. (**D**) Western blot analysis of N protein expression levels in Caco-2–N-WT and Caco-2–N-C28840-Mut stable cell lines. (**E**) qPCR analysis of RNA levels of N, E, and S genes in Caco-2 cells reconstituted with WT N (N-WT) or N-C28840-Mut by lentiviral system, with infection by SARS-CoV-2 GFP/ΔN trVLP at an MOI = 0.05 for 72 hours. (**F**) TCID_50_ assay of SARS-CoV-2 GFP/ΔN trVLP in supernatant from Caco-2 cells reconstituted with WT N (N-WT) or N-C28840-Mut by lentiviral system, with infection by SARS-CoV-2 GFP/ΔN trVLP at an MOI = 0.05 for 72 hours. Data are representative of three independent experiments and were analyzed by two-tailed unpaired *t* test. Graphs show the means ± SD (*n* = 3) derived from three independent experiments. NS, not significant for *P* > 0.05, **P < 0.01, ***P < 0.001.

Coronaviruses are known to generate sgRNAs through TRS-dependent template switching. The N region is located near the 3′ terminus of gRNA. Most of the sgRNAs contain the N region, and the N sgRNA is the most abundant among the various sgRNAs. The results of RNA-Bis-seq (Fig. 2I and fig. S5) and m^5^C-MeRIP-seq (Fig. 1E) both revealed that the N region was highly methylated. Therefore, we chose to further investigate the methylation and biological function of NSUN2 to N. On the one hand, we found that overexpression of NSUN2 inhibited the RNA levels of N, while knockout of NSUN2 elevated the RNA levels of N both in Vero E6 cells and human embryonic kidney (HEK) 293T cells (Fig. 4A and fig. S10A). Reconstitution of WT NSUN2 but not the methyltransferase inactivated mutant (*50*) in NSUN2 knockout cells restored the inhibition function to N RNA levels. Of note, the N transcripts in NSUN2 knockout cells showed higher RNA stability compared to WT cells (Fig. 4B and fig. S10B). Correspondingly, reconstitution of WT NSUN2 but not the methyltransferase inactivated mutant in NSUN2 knockout cells restored the inhibitory function on RNA stability of N transcripts. On the other hand, we developed a BSL-2 cell culture system for production of trVLP, which expresses GFP protein replacing viral N protein (SARS-CoV-2 GFP/ΔN trVLP). We then used the lentiviral system to generate stable WT SARS-CoV-2-N or SARS-CoV-2-N-C28840-mut cell lines in Caco-2 cells, which can be infected by SARS-CoV-2 GFP/ΔN trVLP. By this system, we identified that the SARS-CoV-2 GFP/ΔN trVLP in the SARS-CoV-2-N-C28840-mut Caco-2 cell line showed higher replication level than that in the SARS-CoV-2-N-WT Caco-2 cell line (Fig. 4, C to F). Both intracellular (Fig. 4, C and E) and extracellular viruses (Fig. 4F) were notably elevated in the SARS-CoV-2-N-C28840-mut Caco-2 cell line. Because of the gain of function of SARS-CoV-2 caused by the mutation of the m^5^C methylation sites, reverse genetics cannot be used to make mutant recombinant virus. Together, the results verified that this regulation of N transcripts by NSUN2 was dependent on its m^5^C methyltransferase activity, and the mutation of m^5^C site on N promoted the trVLP replication level.

### The SARS-CoV-2 GFP/ΔN trVLPs with low m^5^C modification showed a stronger replication ability

**Fig. 5.**
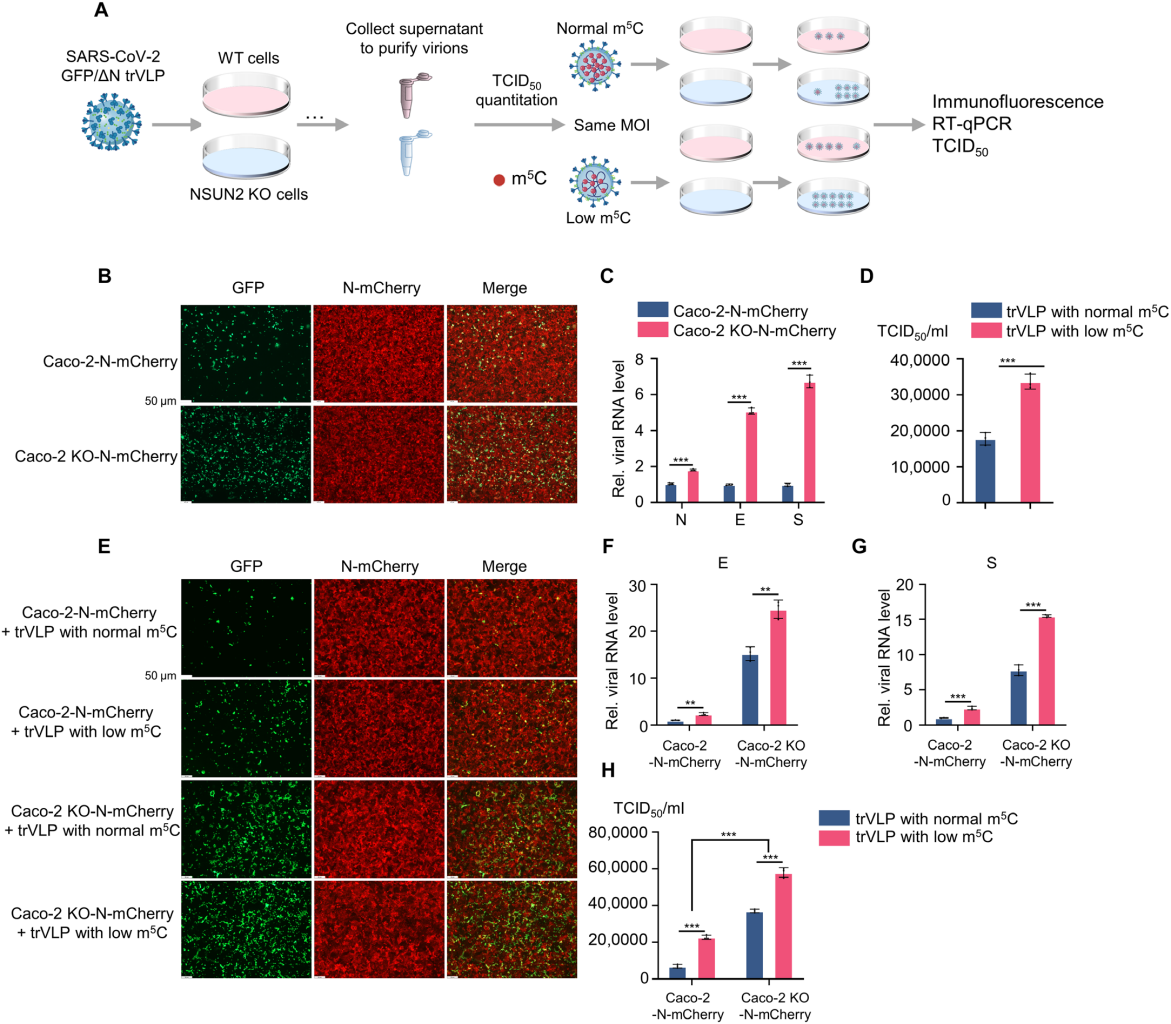
The SARS-CoV-2 GFP/ΔN trVLPs with low m^5^C modification showed a stronger replication ability. (**A**) Schematic diagram of virus amplification and identification of SARS-CoV-2 GFP/ΔN trVLP from WT Caco-2 cells or NSUN2 knockout Caco-2 cells. (**B**) Immunofluorescence analysis of SARS-CoV-2 GFP/ΔN trVLP (green) in WT Caco-2 cells or NSUN2 knockout Caco-2 cells reconstituted with WT N (N-mCherry, red) by lentiviral system, with infection by SARS-CoV-2 GFP/ΔN trVLP at an MOI = 0.05 for 72 hours. Scale bar, 50 μm. (**C**) qPCR analysis of RNA levels of N, E, and S genes in WT Caco-2 cells or NSUN2 knockout Caco-2 cells reconstituted with WT N (N-mCherry, red) by lentiviral system, with infection by SARS-CoV-2 GFP/ΔN trVLP at an MOI = 0.05 for 72 hours. (**D**) TCID_50_ assay of SARS-CoV-2 GFP/ΔN trVLP in supernatant from WT Caco-2 cells or NSUN2 knockout Caco-2 cells reconstituted with WT N (N-mCherry, red) by lentiviral system, with infection by SARS-CoV-2 GFP/ΔN trVLP at an MOI = 0.05 for 72 hour. (**E** to **H**) SARS-CoV-2 GFP/ΔN trVLP with m^5^C or without m^5^C from Fig. 5D was used to infect WT Caco-2 cells or NSUN2 knockout Caco-2 cells reconstituted with WT N (N-mCherry, red) by lentiviral system, at the same MOI. Immunofluorescence analysis (E), qPCR analysis of RNA levels of E and S genes in cell, and TCID_50_ assay of SARS-CoV-2 GFP/ΔN trVLP in supernatant were detected 72 hours after infection. Data are representative of three independent experiments and were analyzed by two-tailed unpaired *t* test. Graphs show the means ± SD (*n* = 3) derived from three independent experiments. NS, not significant for *P* > 0.05, **P < 0.01, ***P < 0.001.

By using the trVLP system, we further explored the effect of the presence or absence of m^5^C modifications by NSUN2 on the replication ability of these trVLPs (Fig. 5A). Consistent with the previous results, knockout of NSUN2 elevated the replication of SARS-CoV-2 GFP/ΔN trVLP both in protein level and RNA level (Fig. 5, B and C). In addition, the median tissue culture infectious dose (TCID_50_) of SARS-CoV-2 GFP/ΔN trVLP amplified from NSUN2 knockout Caco-2 cells (low m^5^C) was higher than that from WT cells (normal m^5^C) (Fig. 5D). Further, we used the SARS-CoV-2 GFP/ΔN trVLP with NSUN2-mediated m^5^C methylation (normal m^5^C) and SARS-CoV-2 GFP/ΔN trVLP without NSUN2-mediated m^5^C methylation (low m^5^C) to infect Caco-2–N-mCherry cells and NSUN2 knockout Caco-2–N-mCherry cells at the same multiplicity of infection (MOI) (Fig. 5A). Strikingly, SARS-CoV-2 GFP/ΔN trVLP without NSUN2-mediated m^5^C methylation showed a stronger replication ability compared to SARS-CoV-2 GFP/ΔN trVLP without NSUN2-mediated m^5^C methylation, both in Caco-2–N-mCherry cells and NSUN2 knockout Caco-2–N-mCherry cells (Fig. 5, E to H). Caco-2–N-mCherry cells infected with SARS-CoV-2 GFP/ΔN trVLP with NSUN2-mediated m^5^C showed the lowest replication ability, while the NSUN2 knockout Caco-2–N-mCherry cells infected with SARS-CoV-2 GFP/ΔN trVLP without NSUN2-mediated m^5^C methylation showed the highest replication ability. Collectively, these results further demonstrated that NSUN2-mediated m^5^C modification negatively regulated SARS-CoV-2 replication, and SARS-CoV-2 GFP/ΔN trVLPs without NSUN2-mediated m^5^C showed a stronger replication ability.

### NSUN2 regulated SARS-CoV-2 replication in two mice models

**Fig. 6.**
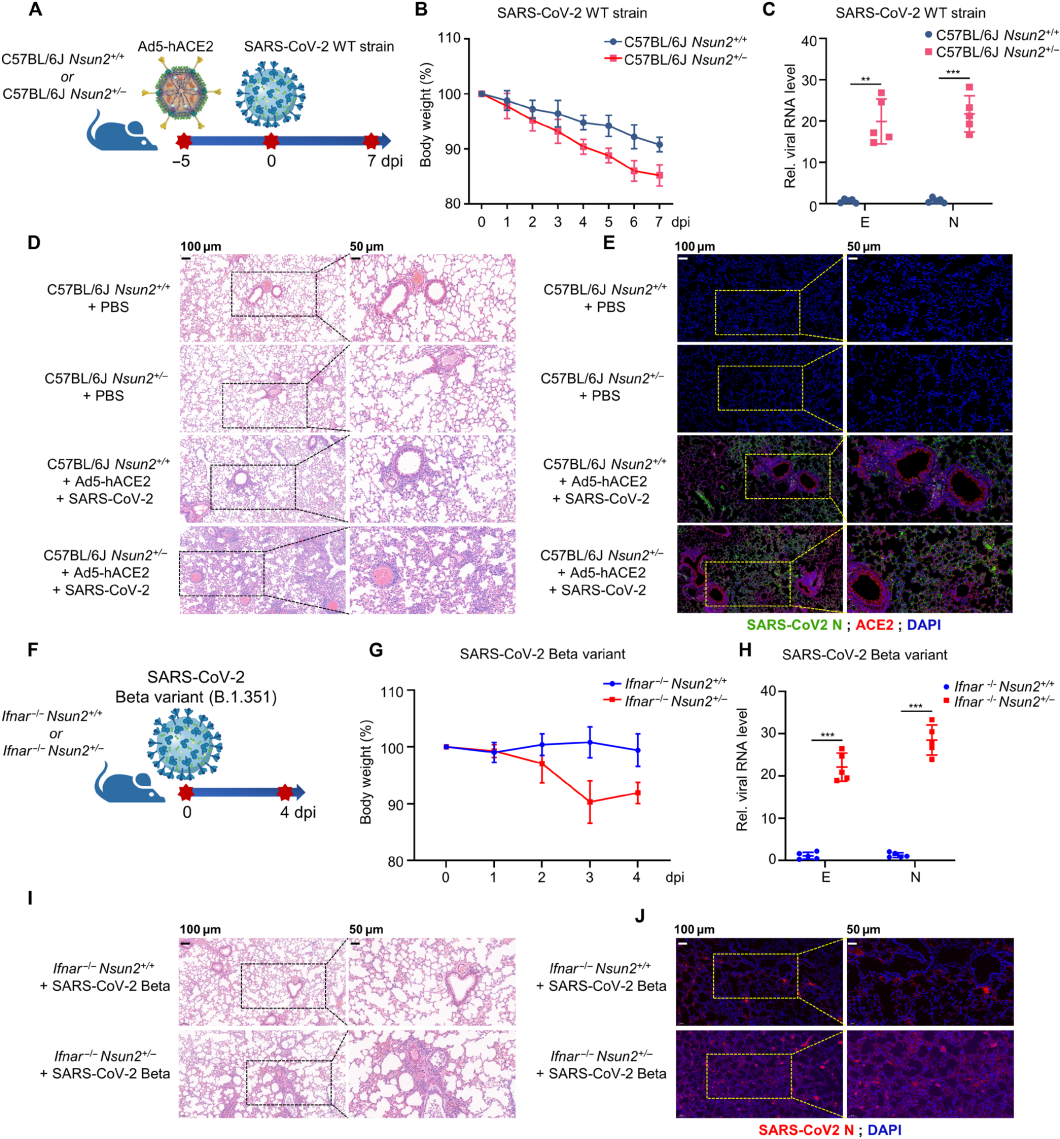
*Nsun2* deficiency mice exhibited higher viral burden and more severe lung tissue damages. (**A**) Timeline of Ad5-hACE2–mediated SARS-CoV-2 WT strain infection mice model (*Nsun2^+/+^* mice and *Nsun2^+/−^* mice). (**B**) Body weight changes of SARS-CoV-2 WT strain–infected *Nsun2^+/+^* mice (*n* = 5) and *Nsun2^+/−^* mice (*n* = 5). (**C**) qPCR analysis of RNA levels of N and E genes in lungs from Ad5-hACE2–mediated SARS-CoV-2 WT strain–infected *Nsun2^+/+^* mice (*n* = 5) or *Nsun2^+/−^* mice (*n* = 5). (**D**) The hematoxylin and eosin (H&E) stain of lungs of uninfected mice group and Ad5-hACE2–mediated SARS-CoV-2 WT strain–infected mice group (*Nsun2^+/+^* mice and *Nsun2^+/−^* mice). (**E**) Immunofluorescence analysis of Ad5-hACE2–mediated SARS-CoV-2 WT strain–infected mice group (*Nsun2^+/+^* mice and *Nsun2^+/−^* mice). SARS-CoV-2 N (green). ACE2 (red). 4′,6-diamidino-2-phenylindole (DAPI, blue). (**F**) Timeline of SARS-CoV-2 Beta variant (B.1.351) infection mice model (*Ifnar^−/−^Nsun2^+/+^* mice and *Ifnar^−/−^Nsun2^+/−^* mice). (**G**) Body weight changes of SARS-CoV-2 Beta variant (B.1.351)–infected *Ifnar^−/−^Nsun2^+/+^* mice (*n* = 5) and *Ifnar^−/−^Nsun2^+/−^* mice (*n* = 5). (**H**) qPCR analysis of RNA levels of N and E genes in lungs from SARS-CoV-2 Beta variant (B.1.351)–infected *Ifnar^−/−^Nsun2^+/+^* mice (*n* = 5) and *Ifnar^−/−^Nsun2^+/−^* mice (*n* = 5). (**I**) The H&E stain of lungs of SARS-CoV-2 Beta variant (B.1.351)–infected *Ifnar^−/−^Nsun2^+/+^* mice and *Ifnar^−/−^Nsun2^+/−^* mice. (**J**) Immunofluorescence analysis of lungs of SARS-CoV-2 Beta variant (B.1.351)–infected *Ifnar^−/−^Nsun2^+/+^* mice and *Ifnar^−/−^Nsun2^+/−^* mice. SARS-CoV-2 N (red). DAPI (blue). **P < 0.01, ***P < 0.001.

To further investigate the role of NSUN2 in the regulation of SARS-CoV-2 infection in vivo, we created targeted deletions of NSUN2 in mice by removing 10 base pairs (bps) in exon 3 of *Nsun2* genome by CRISPR-Cas9, which resulted in a frameshift mutation (*50*). However, the homozygous *Nsun2^−/−^* mice died in utero. Therefore, we chose *Nsun2^+/−^* mice as *Nsun2* deficiency mice (fig. S11). Then, we used an adenoviral transduction–based mouse model, which can be infected with SARS-CoV-2 in C57BL/6J mice (Fig. 6A) (*51*). *Nsun2^+/+^* and *Nsun2^+/−^* mice were anesthetized with isoflurane and intranasally transduced with Ad5-hACE2. Five days after transduction, the mice were then infected intranasally with SARS-CoV-2. The *Nsun2^+/−^* mice lost more weight than the *Nsun2^+/+^* mice (Fig. 6B), and there was a higher viral burden of SARS-CoV-2 in lungs of *Nsun2^+/−^* mice than in WT mice at the RNA levels of N and E (Fig. 6C). Meanwhile, *Nsun2^+/−^* mice showed a higher viral burden of SARS-CoV-2 (N protein) in lungs and more severe lung tissue damages compared with their WT littermates (Fig. 6, D and E).

To block the effect of interferon responses induced by NSUN2 deletion, we crossed the *Nsun2^+/−^* mice with *Ifnar^−/−^* and got the *Ifnar^−/−^Nsun2^+/+^* mice and *Ifnar^−/−^Nsun2^+/−^* mice. Then, we used SARS-CoV-2 Beta variant (B.1.351) infection mice model to further verify the NSUN2-mediated regulation of SARS-CoV-2 infection in vivo (Fig. 6F)*.* The *Ifnar^−/−^Nsun2^+/−^* mice lost up to about 10% of their weight in the first 3 to 4 days of infection, while the *Ifnar^−/−^Nsun2^+/+^* mice did not show notable weight change (Fig. 6G). Accordingly, *Ifnar^−/−^Nsun2^+/−^* mice showed a higher viral burden of SARS-CoV-2 in lungs and more severe lung tissue damages compared with *Ifnar^−/−^Nsun2^+/+^* mice both at the protein levels and RNA levels (Fig. 6, H to J). Together, the results of the two SARS-CoV-2–infected mice models suggest that NSUN2 was a negative regulator of SARS-CoV-2 and quite pivotal for the replication and pathogenicity of SARS-CoV-2 in vivo, which may serve as a strategy for the host to resist the replication of SARS-CoV-2.

### NSUN2 expression was antagonized by SARS-CoV-2 infection

**Fig. 7.**
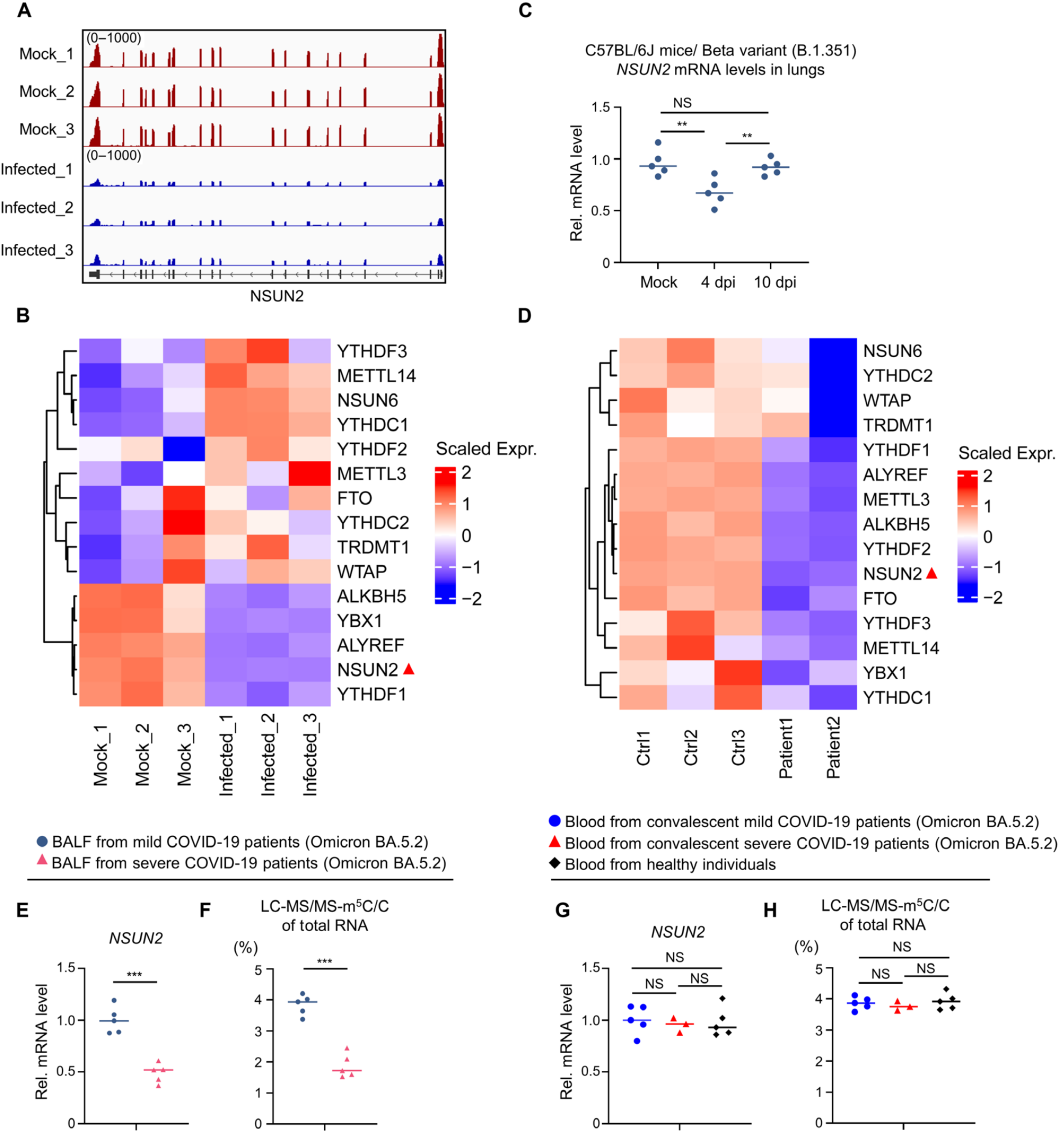
NSUN2 expression decreased during SARS-CoV-2 infection. (**A**) RNA-seq signals for NSUN2 mRNA in uninfected Caco-2 cells (Mock) and SARS-CoV-2 (WT strain, 24 hours)–infected Caco-2 cells (Infected). The scale on the *y* axis indicates the read density per million of total normalized reads. (**B**) Heatmap showed the expression levels of m^5^C and m^6^A machinery genes in uninfected Caco-2 cells (Mock) and SARS-CoV-2–infected Caco-2 cells (Infected). (**C**) qPCR analysis of mRNA levels of *Nsun2* in lungs from the uninfected C57BL/6J mice, the C57BL/6J mice infected with SARS-CoV-2 Beta variant (B.1.351) for 4 days with a high viral load, and the C57BL/6J mice infected with SARS-CoV-2 Beta variant (B.1.351) for 10 days with a low viral load. (**D**) Heatmap showed the expression levels of m^5^C and m^6^A machinery genes in BALF of patients with COVID-19 (Patient1 and Patient2) and healthy controls (Ctrl1, Ctrl2, and Ctrl3). Data were obtained from the analysis of our previous results (*52*). (**E**) qPCR analysis of RNA levels of NSUN2 in BALF from seriously ill patients (*n* = 5) and mildly ill patients (*n* = 5) with the SARS-CoV-2 Omicron subvariant BA.5.2. (**F**) Analysis of m^5^C/C ratio using LC-MS/MS of BALF from seriously ill (*n* = 5) and mildly ill patients with COVID-19 (*n* = 5). (**G**) qPCR analysis of RNA levels of NSUN2 in blood from convalescent patients with severe COVID-19 (*n* = 5) and convalescent patients with mild COVID-19 (*n* = 5) with the SARS-CoV-2 Omicron subvariant BA.5.2. (**H**) Analysis of m^5^C/C ratio using LC-MS/MS of blood from the convalescent patients with severe COVID-19 (*n* = 3), the convalescent patients with mild COVID-19 (*n* = 5), and the healthy individuals (*n* = 5). NS, not significant for *P* > 0.05, **P < 0.01, ***P < 0.001.

To investigate the biological role of NSUN2 during SARS-CoV-2 infection, we performed RNA sequencing (RNA-seq) to systematically analyze the expression levels of mRNAs in Caco-2 cells or Caco-2 cells with SARS-CoV-2 infection for 24 hours. *NSUN2* mRNA levels were consistently decreased in SARS-CoV-2–infected Caco-2 cells compared to mock Caco-2 cells (Fig. 7, A and B). Using the SARS-CoV-2 Beta variant (B.1.351)–infected mouse model, we found that endogenous *Nsun2* mRNA levels also decreased in SARS-CoV-2 Beta variant (B.1.351)–infected group [4 days post infection (dpi)] compared to the uninfected group (Mock) (Fig. 7C). However, there was no notable difference in the endogenous *Nsun2* mRNA levels between the infected group (10 dpi) and the uninfected group. According to our results, the SARS-CoV-2 Beta variant (B.1.351)–infected mouse model showed mild symptoms, and, in the infected group (10 dpi), the mice regained weight and recovered with low viral loads in their lung tissues. These results suggested that when SARS-CoV-2 replicates to a higher level, it suppresses the expression level of NSUN2 through some unknown mechanism. In addition, we carried out transcriptome sequencing of the RNAs isolated from the bronchoalveolar lavage fluid (BALF) of two patients with COVID-19. *NSUN2* mRNA levels were also reduced in patients with COVID-19 compared with healthy individuals (Fig. 7D) (*52*). From our above results, NSUN2 negatively regulated the replication of SARS-CoV-2 via epitranscriptomic addition of m^5^C modification to SARS-CoV-2 transcripts. This mechanism may be exploited by the host as an antiviral strategy. Therefore, SARS-CoV-2 will antagonize NSUN2 expression via unknown mechanism. We have systematically screened the proteins encoded by SARS-CoV-2 but did not find a specific regulatory factor (fig. S12A). Viral infections will induce the production of interferons and proinflammatory factors as a common feature. Therefore, we speculated whether interferons or proinflammatory factors decreased NSUN2 expression. Then, we screened interferon-β (IFN-β), tumor necrosis factor–α (TNF-α), and interleukin-1β (fig. S12B). However, NSUN2 mRNA levels did not show notable change with treatment of these factors. On the basis of the current data, we cannot give a clear mechanism at this time. We will further explore the specific mechanism of this degradation in follow-up work. The expression levels of the two m^5^C readers, ALYREF and YBX1, also decreased upon SARS-CoV-2 infection, although they were not involved in the regulation of SARS-CoV-2 (Fig. 2A). Still, this result may uncover that SARS-CoV-2 infection will have an impact on host m^5^C machinery and m^5^C methylome. However, the expression levels of m^6^A machinery genes did not show notable or consistent change compared with m^5^C machinery (Fig. 7, B and D). We further collected BALF from patients with severe and mild COVID-19 with the SARS-CoV-2 Omicron subvariant BA.5.2 and found that NSUN2 expression levels and m^5^C levels decreased in patients with severe compared to mild COVID-19 (Fig. 7, E and F). Unfortunately, two of the patients with severe COVID-19 passed away. We then collected the blood of these convalescent patients. The NSUN2 expression levels and m^5^C levels of these convalescent patients restored to the same level as in healthy individuals (Fig. 7, G and H), which is consistent with the above results of SARS-CoV-2 Beta variant (B.1.351)–infected mouse model (Fig. 7C). These results provide further evidence that m^5^C machinery is involved in the regulatory process of SARS-CoV-2 replication and NSUN2 expression was antagonized by SARS-CoV-2 infection via unknown mechanism.

We further measured different time points to understand the kinetics of m^5^C modification mediated by NSUN2. NSUN2 expression levels gradually decreased upon SARS-CoV-2 infection, while the m^5^C levels of total RNAs gradually increased to the highest value and then decrease (fig. S13). Because NSUN2 is constitutively expressed in uninfected cells, in the early stage of viral infection, the level of NSUN2 is relatively abundant and enough to modify large amounts of the newborn SARS-CoV-2 RNAs. Therefore, the m^5^C levels of total RNAs will gradually increase because of the newborn m^5^C-modified SARS-CoV-2 RNAs. However, in the late stages of viral infection, because of the decrease of NSUN2 caused by SARS-CoV-2 infection, there are fewer NSUN2 proteins in the cell, and large amounts of progeny viruses containing m^5^C modifications are released outside the cell. Then, the m^5^C modification level of total RNAs will begin to decline.

### The SARS-CoV-2 virions with low m^5^C modification from *Nsun2^+/−^* mice exhibited a stronger replication ability and caused more severe lung tissue damages in K18-hACE2 mice

**Fig. 8.**
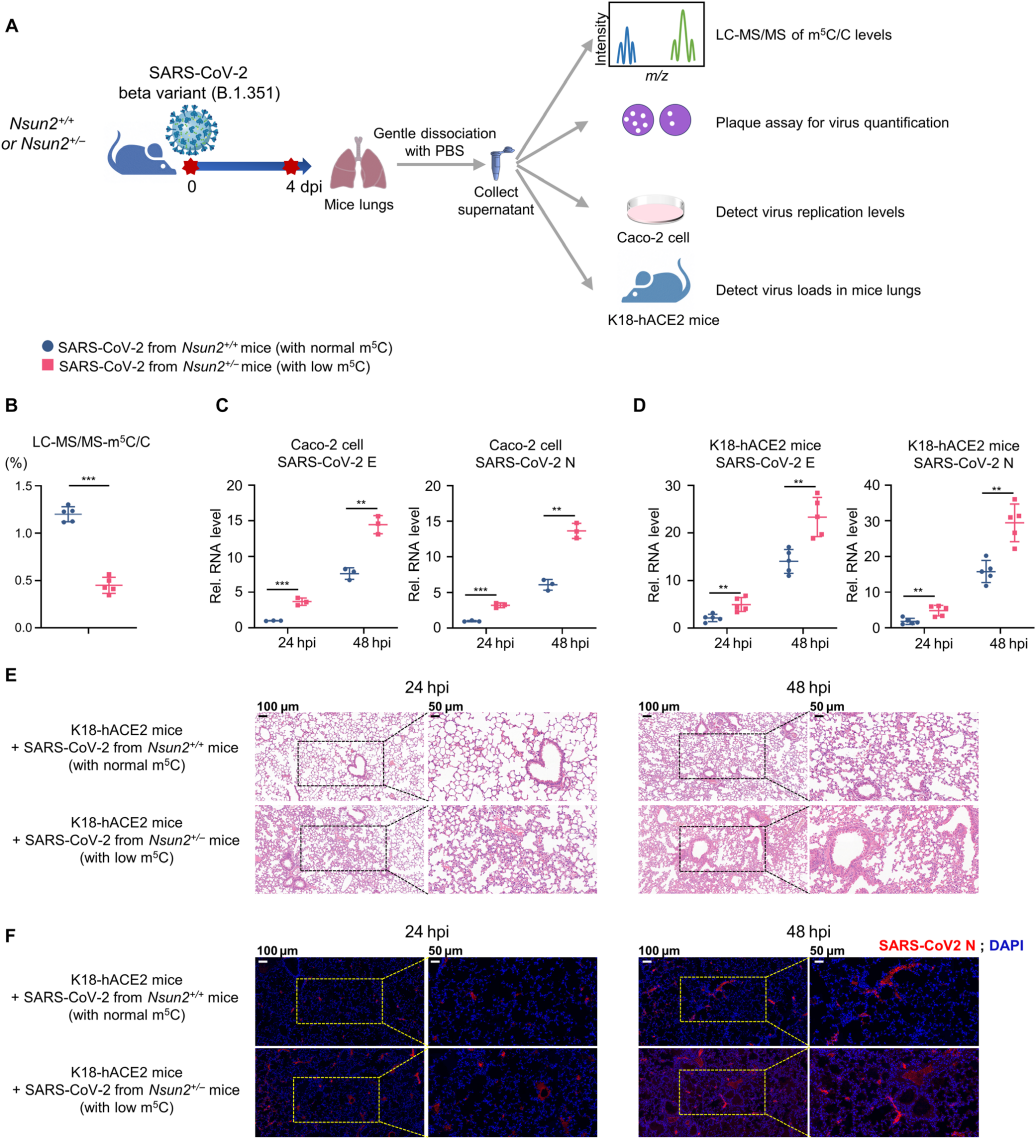
The progeny SARS-CoV-2 virions from *Nsun2^+/−^* mice with low m^5^C modification showed a stronger replication ability in Caco-2 cells and K18-hACE2 mice. (**A**) Schematic diagram of virus amplification and identification of SARS-CoV-2 virions from *Nsun2^+/+^* mice or *Nsun2^+/−^* mice. (**B**) Analysis of m^5^C/C ratio using LC-MS/MS of SARS-CoV-2 virion RNA from lungs of Beta variant (B.1.351)–infected *Nsun2^+/+^* mice (*n* = 5) or *Nsun2^+/−^* mice (*n* = 5). (**C**) The purified SARS-CoV-2 virions from lungs of Beta variant (B.1.351)–infected *Nsun2^+/+^* mice or *Nsun2^+/−^* mice were further used to infect Caco-2 cells at the same MOI for 24 and 48 hours, followed by qPCR analysis of RNA levels of E and N genes from the infected Caco-2 cells. (**D** to **F**) The purified SARS-CoV-2 virions from lungs of Beta variant (B.1.351)–infected *Nsun2^+/+^* mice or *Nsun2^+/−^* mice were further used to infect K18-hACE2 mice (*n* = 5) at the same MOI for 24 and 48 hours, followed by qPCR analysis (D) of RNA levels of E and N genes, H&E stain of lungs (E), and immunofluorescence analysis of lungs (F) from the infected K18-hACE2 mice. SARS-CoV-2 N (red). DAPI (blue). **P < 0.01, ***P < 0.001.

To further verify the differences in the replication ability and virulence of SARS-CoV-2 virions with normal or low m^5^C modification in vivo, we used SARS-CoV-2 Beta variant (B.1.351) to infect *Nsun2^+/+^* mice and *Nsun2^+/−^* mice, respectively, and gently dissociated the lungs to collect the progeny SARS-CoV-2 virions from *Nsun2^+/+^* mice or *Nsun2^+/−^* mice (Fig. 8A). We quantified the progeny SARS-CoV-2 virions from *Nsun2^+/+^* mice or *Nsun2^+/−^* mice, analyzed the m^5^C/C ratio using LC-MS/MS of the progeny SARS-CoV-2 virions, and then used them to infect Caco-2 cells and K18-hACE2 mice, respectively. The LC-MS/MS results showed that the m^5^C level of the progeny SARS-CoV-2 virions from *Nsun2^+/−^* mice reduced by about half (Fig. 8B). Consistent with the previous SARS-CoV-2 GFP/ΔN trVLP results in Fig. 5, the progeny SARS-CoV-2 virions from *Nsun2^+/−^* mice (with low m^5^C level) showed a stronger replication ability both in Caco-2 cells (Fig. 8C) and K18-hACE2 mice (Fig. 8D) than the progeny SARS-CoV-2 virions from *Nsun2^+/+^* mice (with normal m^5^C level). Meanwhile, the progeny SARS-CoV-2 virions from *Nsun2^+/−^* mice (with low m^5^C level) caused more severe lung tissue damages in K18-hACE2 mice and a higher viral burden of SARS-CoV-2 (N protein) in lungs (Fig. 8, E and F).

Together, these results provide strong evidence that m^5^C modification is an important way to regulate SARS-CoV-2 replication and virulence, and the progeny SARS-CoV-2 virions with low m^5^C modification replicates more strongly inside the host in vivo*.* NSUN2-mediated m^5^C methylation of SARS-CoV-2 RNA negatively regulates viral replication and the virulence of progeny viruses in the new infection.

## DISCUSSION

Inconsistent results of the m^6^A-mediated regulation of SARS-CoV-2 replication have been reported. Two groups reported that m^6^A RNA methylation negatively regulates the SARS-CoV-2 life cycle (*36*, *37*), while three groups demonstrated that m^6^A positively regulates SARS-CoV-2 replication (*38*–*40*). We found that knockdown of m^6^A writers mildly increased SARS-CoV-2 replication (Fig. 2A). However, knockdown or knockout of m^5^C writers NSUN2 notably increased SARS-CoV-2 replication. Moreover, according to our results of LC-MS/MS, m^5^C is more abundant than m^6^A in SARS-CoV-2 virion RNA (Fig. 1D and fig. S1). Together, the above results demonstrate that m^5^C RNA methylation showed more notable regulation of SARS-CoV-2 than m^6^A.

From the results of RNA-Bis-seq and m^5^C-MeRIP-seq (Figs. 1E and 2I and fig. S5), the m^5^C methylation sites were distributed across the entire gRNA, with no particularly hypermethylated m^5^C methylation sites. The Input signal and m^5^C-IP signal near the N region are very high because of the highest expression level of N sgRNAs, but the ratios of m^5^C-IP/Input in N region or other m^5^C-modified regions are close. The N gene contains NSUN2-dependent m^5^C sites, which play notable roles in regulating the function of N expression and the replication of SARS-CoV-2. Meanwhile, the other m^5^C sites were also identified in other regions, such as nsp14, nsp15, nsp16, E, M, and S. The m^5^C incorporation experiment suggested that m^5^C modification had a notable effect on the degradation of viral RNA fragments as a whole effect (Fig. 3C and fig. S8). The identified m^5^C methylation sites among nsp14, nsp15, nsp16, S, E, M, and N were proved to be functional in regulating SARS-CoV-2 RNA stability, and the mutations of these cytosines enhance RNA stability. However, we found that the mutation of the three high methylation sites in ORF3a decreased RNA expression levels, unlike nsp14, nsp15, nsp16, S, E, M, and N. We found that the methylation sites on ORF3a had different regulatory mechanisms from other regions. We are conducting further research to investigate this phenomenon.

Our previous work revealed that NSUN2 serves as a negative regulator of type I interferon responses in antiviral innate immunity during various viral infections (*50*). Knockout of NSUN2 can enhance type I interferon responses and downstream interferon-stimulated genes (ISGs) expression after some viral infections both in vitro and in vivo, thus inhibiting the replication of vesicular stomatitis virus (VSV), herpes simplex virus 1 (HSV-1), or Sendai virus (SeV). However, the interferon responses caused by SARS-CoV-2 infection is not so strong as VSV, HSV-1, and SeV. SARS-CoV-2 invests substantial resources to block the establishment of the antiviral response (*53*). Therefore, the enhanced interferon responses by NSUN2 knockout were not so strong in SARS-CoV-2–infected models. Of note, in this work, the regulation of NSUN2 on SARS-CoV-2 is independent of interferon responses but by directly affecting the stability of viral RNAs. This suggests that the direct effect of NSUN2 on SARS-CoV-2 is much greater than the effect on interferon responses during SARS-CoV-2 infection, unlike some other viruses.

The currently known readers of RNA m^5^C modification are not involved in regulating the replication of SARS-CoV-2 (Fig. 2A). The reason may be the irrelevance of their functions. ALYREF was reported to promote the export of m^5^C-modified mRNAs (*47*). However, SARS-CoV-2 replicates only in the cytoplasm. YBX1 maintains the stability of its target mRNA with m^5^C modification (*48*), but our results demonstrated that NSUN2 mediated the methylation of SARS-CoV-2 RNA transcripts and facilitated their degradation. These results imply the involvement of unidentified m^5^C reader proteins that may contribute to the regulation of SARS-CoV-2 replication. The specific degradation mechanism induced by m^5^C modification has not yet been clarified clearly and requires further investigation. Another study reported that m^5^C modification of Epstein-Barr virus RNA decreases its stability (*46*), which may also support the host antiviral strategy we propose via epitranscriptomic addition of m^5^C methylation to viral RNAs. Further work is required to delineate these different mechanisms and roles that different m^5^C readers play in the regulation of various viruses.

**Fig. 9.**
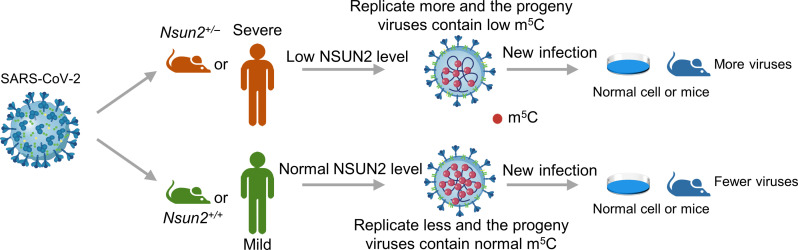
Schematic diagram of NSUN2-mediated m^5^C modification regulates SARS-CoV-2 replication and the virulence of progeny viruses in the new infection*.* We used a mouse model to simulate patients with severe and mild COVID-19 and revealed that progeny SARS-CoV-2 virions from *Nsun2^+/−^* mice (with low m^5^C level, simulating patients with severe COVID-19) showed a stronger replication ability and virulence in the new round of infection in a K18-hACE2 mouse model than the progeny SARS-CoV-2 virions from *Nsun2^+/+^* mice (with normal m^5^C level, simulating patients with mild COVID-19 or healthy individuals).

Since SARS-CoV-2 itself does not encode the NSUN2 protein, the original SARS-CoV-2 RNA may not contain so many m^5^C modifications. According to our results (Fig. 5), SARS-CoV-2 GFP/ΔN trVLP without NSUN2-mediated m^5^C methylation showed a stronger replication ability compared to SARS-CoV-2 GFP/ΔN trVLP without NSUN2-mediated m^5^C methylation. We propose a SARS-CoV-2–host interaction model. After SARS-CoV-2 infects the host, the gRNA and sgRNAs of SARS-CoV-2 will be modified with m^5^C modification gradually by the host methyltransferase NSUN2. The m^5^C modification can be recognized by the host as a “marker” and degraded by host factors, thus serving as a host antiviral strategy. This marker is quickly recognized and degraded when the progeny viruses with m^5^C modification enters another cell in the new round of infection. From our results (Fig. 7, D to F), the NSUN2 expression level in patients with severe COVID-19 was lower than that in patients with mild COVID-19. Correspondingly, m^5^C modification on viral and host RNA was reduced in patients with severe COVID-19. When the patients recovered, the NSUN2 expression level in patients with COVID-19 returned to normal as in healthy people (Fig. 7, G and H). Because it is not possible to conduct experiments on reinfection of SARS-CoV-2 (with low or normal m^5^C modification) in humans, we used a mouse model to simulate patients with severe and mild COVID-19 and revealed that progeny SARS-CoV-2 virions from *Nsun2^+/−^* mice (with low m^5^C level, simulating patients with severe COVID-19) showed a stronger replication ability and virulence in the new round of infection in a K18-hACE2 mouse model (Fig. 8) than the progeny SARS-CoV-2 virions from *Nsun2^+/+^* mice (with normal m^5^C level, simulating patients with mild COVID-19 or healthy individuals). The above results may imply that the progeny SARS-CoV-2 viruses transmitted by patients with severe COVID-19 have a stronger replication ability and virulence in the new round of infection when spreading from person to person due to the lack of m^5^C RNA modification (Fig. 9). The mechanism of NSUN2 expression decline caused by SARS-CoV-2 remains to be further investigated, and we are conducting follow-up investigations.

In conclusion, our investigation has revealed a role for NSUN2-mediated m^5^C modification in regulating SARS-CoV-2 replication and virulence. We outline a paradigm that the host has evolved an antiviral strategy via epitranscriptomic addition of m^5^C methylation to SARS-CoV-2 RNAs, which will help in the development of anti-coronavirus drugs or efficient therapeutic interventions.

## MATERIALS AND METHODS

### Viruses, cells, and reagents

The SARS-CoV-2 WT strain (IVCAS 6.7512) and Beta variant (B.1.351) (NPRC2.062100001) were provided by the National Virus Resource, Wuhan Institute of Virology, Chinese Academy of Sciences. Caco-2, Vero E6, HEK293T, and Huh7 cells were maintained in Dulbecco’s modified Eagle’s medium (DMEM) supplemented with 10% fetal bovine serum (FBS), penicillin (100 U/ml), and streptomycin (100 μg/ml) at 37°C in 5% CO_2_ incubator. Plasmids were transfected using Lipofectamine 3000 (Invitrogen) or Neofect DNA transfection reagent (Neofect, TF201201) following the manufacturer’s instructions, and siRNAs (RiboBio, Guangzhou, China) were transfected using RNAiMAX (Invitrogen) following the manufacturer’s instructions. Ruxolitinib and actinomycin D were purchased from MCE (MedChemExpress).

### Mice

*Nsun2^+/−^* C57BL/6J mice were obtained from Gempharmatech Co. Ltd. (Nanjing, China) and housed and bred in specific pathogen–free conditions. The primers used for genotyping of *Nsun2* mutant mice were F-TGTCCAACAGAACAGTGAACTGGAG and R-CCAAGCTCTTTAAGCCGACAGTG. *Ifnar^−/−^* mice were a gift from B. Zhong (Wuhan University). The primers used for genotyping of *Ifnar^−/−^* mice were F-CGAGGCGAAGTGGTTAAAAG, R1-ACGGATCAACCTCATTCCAC (WT reverse primer), and R2-AATTCGCCAATGACAAGACG (mutant reverse primer). *Nsun2^+/−^* C57BL/6J mice were crossed with *Ifnar^−/−^* mice to obtain *Ifnar^−/−^Nsun2^+/+^* mice and *Ifnar^−/−^Nsun2^+/−^* mice. All animal experiments were conducted in accordance with the Regulations of Hubei Province Laboratory Animal Management and approved by Wuhan University Animal Experiment Ethics Committee.

### SARS-CoV-2 live virus infection assay

All SARS-CoV-2 live virus–related experiments were approved by the level 3 Biosafety Committee (ABSL-3) of Wuhan University. All experiments involving SARS-CoV-2 were performed in the BSL-3 and ABSL-3 facilities of Wuhan University.

### SARS-CoV-2 infection mouse models

The establishment of Ad5-hACE2 mouse model was performed as described previously (*51*). Briefly, 8-week-old *Nsun2^+/+^* C57BL/6J mice and *Nsun2^+/−^* C57BL/6J mice were lightly anesthetized with isoflurane and transduced intranasally with 2.5 × 10^8^ focus-forming units of Ad5-hACE2 in 75 μl of DMEM. Five days after transduction, the mice were infected intranasally with 1 × 10^5^ plaque-forming units (PFU) of SARS-CoV-2 WT strain (IVCAS 6.7512) in 50 μl of DMEM. The mice were monitored and weighted daily. Seven days after infection, the mice were euthanized and lung tissues were taken.

For the establishment of SARS-CoV-2 Beta variant (B.1.351) (NPRC2.062100001) infection mouse model, 8-week-old *Ifnar^−/−^Nsun2^+/+^* mice and *Ifnar^−/−^Nsun2^+/−^* mice were lightly anesthetized with isoflurane and intranasally with 1.25 × 10^5^ PFU of SARS-CoV-2 Beta variant (B.1.351) in 50 μl of DMEM. The mice were monitored and weighted daily. Four days after infection, the mice were euthanized and lung tissues were taken.

### Histopathological analysis and immunofluorescence assay for mice tissues

Histopathological analysis and immunofluorescence assay for mice tissues were performed by Wuhan Servicebio Technology Co. Ltd. For histopathological analysis, the mice tissues were immediately fixed in 4% paraformaldehyde (PFA), embedded in paraffin, sectioned, and stained with hematoxylin and eosin (H&E). For immunofluorescence assay, the sections were blocked with phosphate-buffered saline (PBS) containing 3% bovine serum albumin (BSA) at room temperature for 30 min and then incubated with primary antibody (diluted with PBS appropriately) overnight at 4°C. After being washed three times with PBS, the slides were incubated with fluorescent-labeled secondary antibody (appropriately respond to primary antibody in species) at room temperature for 50 min in dark condition. After being washed for another three times with PBS, the slides were incubated with 4′,6-diamidino-2-phenylindole (DAPI) solution at room temperature for 10 min in dark condition.

### Antibodies and immunoblot analysis

The antibodies used were as follows: rabbit anti-NSUN2 (Proteintech, 20854-1-AP), rabbit anti-METTL3 (Proteintech, 15073-1-AP), mouse anti–SARS-CoV-2 N (Sino Biological, 40143-MM05), rabbit anti-human ACE2 (Sino Biological, 10108-RP01), and mouse anti–glyceraldehyde-3-phosphate dehydrogenase (GAPDH, Proteintech, 60004-1-Ig). The cells were washed once with PBS and lysed in radioimmunoprecipitation assay or NP-40 lysis buffer (Beyotime). SDS loading buffer (5×) was added to the protein sample and boiled for 10 min. The samples were resolved on SDS–polyacrylamide gel electrophoresis and transferred onto nitrocellulose membrane (GE Healthcare), followed by blocking with tris-buffered saline containing 0.1% Tween-20 (TBST) and 5% nonfat powdered milk or BSA and probing with different antibodies.

### Immunofluorescence assay

Caco-2 or Vero E6 cells were seeded in 48-well plates and infected with SARS-CoV-2 for 24 hours. The cells were inactivated and fixed with 4% PFA at room temperature for 30 min, permeabilized with 0.2% Triton X-100 at room temperature for 10 min, and blocked with 1% BSA at 37°C for 30 min. Then, the cells were incubated with SARS-CoV-2 N antibody (40143-MM05, Sino Biological) diluted in 1% BSA at 37°C for 1 hour, followed by PBS washing. Next, the cells were incubated with Alexa Fluor 594–conjugated goat anti-mouse immunoglobulin G (Thermo Fisher Scientific, A11032) diluted in 1% BSA at room temperature for 30 min, followed by PBS washing. The nucleus was stained with Hoechst 33342 for 5 min. The images were captured with an inverted fluorescence microscope.

### RNA isolation and qPCR

Total RNA was isolated using TRIzol reagent (Invitrogen) following the manufacturer’s instructions. The isolated RNA was reverse transcribed to cDNA using PrimeScript RT Reagent Kit (Takara, RR037A) or NovoScript Plus All-in-one first-strand cDNA Synthesis SuperMix (Novoprotein). Real-time qPCR was carried out through ABI 7500 Real Time PCR System by SYBR Green Master Mix (YEASEN, 11199ES03) or Taqman Probe Master Mix (YEASEN, 11205ES08). GAPDH was used in normalization via the ΔΔCt method. Primer sequences are shown in table S1.

### Liquid chromatography tandem MS

LC-MS/MS was performed by Wuhan Metware Biotechnology Co. Ltd. Briefly, the digestion mixture contains 1 μg of RNA, 1 U of nuclease P1, 10 mM NaCl, and 2 mM ZnCl2 in a final volume of 30 μl. The mixture was incubated at 37°C for 3 hours. Then, 1 U of shrimp alkaline phosphatase and 2.5 μl of ammonium bicarbonate (1 M) were added into the mixture for another 2 hours and diluted to 100 μl. Three microliters of the mixture was injected into the LC-MS/MS. The nucleosides were separated by a C18 column and detected by triple-quadrupole MS (Shimadzu MS-8050 mass spectrometer, Tokyo, Japan). RNA modifications contents were detected by Wuhan Metware Biotechnology Co. Ltd. (http://metware.cn/) based on the AB Sciex QTRAP 6500 LC-MS/MS platform.

### Construction of knockout cell line by CRISPR-Cas9

The gRNAs were NSUN2-gRNA-1: F-CACCGACGCGGAGGATGGCGCCGA and R-AAACTCGGCGCCATCCTCCGCGTC; NSUN2-gRNA-2: F-CACCACCGTG GCGTTTCAGCGGTT and R-AAACAACCGCTGAAACGCCACGGT. The gRNAs were constructed in lentiCRISPR-v2 plasmid (Addgene). Lentiviral backbone (1 μg), psPAX2 (0.5 μg), and pMD2.G (0.5 μg) were transfected into HEK293T cells, which were seeded on six-well plates using Neofect. Forty-eight hours later, supernatants were collected and filtered using a 0.45-μm filter to infect target cells with polybrene (8 μg/ml). The cells were infected twice to get higher transduction efficiency. Then, puromycin was used to screen positive cells, followed by seeding into 96-well plates (1 cell per well). After 2 weeks’ cultivation, single clones were selected following enlarged cultivation with puromycin selection. Single clones were identified by immunoblot analysis, and genomic DNA was extracted followed by PCR and sequencing.

### In vitro transcription assays

RNA was in vitro transcribed with CTP or m^5^CTP (Syngenebio, Nanjing, China) as substrates using a MEGAscript T7 Kit (Ambion, USA) according to the manufacturer’s instructions. The transcribed RNAs were then capped with the Vaccinia Capping Enzyme (NEB, M2080) and mRNA Cap 2′-*O*-methyltransferase (NEB, M0366) in the presence of guanosine triphosphate and SAM if the RNAs were used for transfection.

### In vitro methylation assays

Reaction mixtures (50 μl) containing 0.2 nM recombinant GST–tagged NSUN2, 0.01 nM in vitro–transcribed fragments of mRNA, 1 μCi of *S*-adenosyl [methyl-^3^H] methionine (0.5 μCi/μl, PerkinElmer) in reaction buffer [500 mM tris-HCl (pH 7.5), 5 mM EDTA, 10 mM dithiothreitol, 20 mM MgCl_2_], and 40 U of ribonuclease inhibitor were incubated for 60 min at 37°C. The ^3^H-labeled products were isolated using DEAE-Sephadex A-50 columns and quantitated by liquid scintillation counting (PerkinElmer).

### MeRIP-seq

The m^5^C-MeRIP-seq was provided by Cloudseq Biotech Inc. (Shanghai, China). Briefly, m^5^C RNA immunoprecipitation was performed with the GenSeq m^5^C RNA IP Kit (GenSeq, China) by following the manufacturer’s instructions. Both the input samples without immunoprecipitation and the m^5^C IP samples were used for RNA-seq library construction with NEBNext Ultra II Directional RNA Library Prep Kit (NEB, USA). The library quality was evaluated with BioAnalyzer 2100 system (Agilent Technologies, USA). Library sequencing was performed on an illumina Novaseq 6000 instrument with 150-bp paired-end reads. The m^5^C-MeRIP-seq data have been deposited in the GSA database under the accession number: HRA002087.

### RNA-Bis-seq

The RNA-Bis-seq was provided by Cloudseq Biotech Inc. (Shanghai, China). Briefly, total RNAs for each sample was ribosomal RNA (rRNA)–depleted using GenSeq rRNA Removal Kit (GenSeq Inc.). rRNA-depleted RNA was bisulfite converted and purified using the EZ RNA methylation Kit (Zymo Research). RNA libraries were then constructed with GenSeq Low Input RNA Library Prep Kit (GenSeq Inc.) according to the manufacturer’s instructions. The library was quality-controlled with BioAnalyzer 2100 system (Agilent Technologies Inc.) and then sequenced in an Illumina Novaseq 6000 instrument. Analysis of the spike-in showed C to T conversion rates >99%. The RNA-Bis-seq data have been deposited in the GSA database under the accession number (HRA006230).

### RNA sequencing

Total RNAs were extracted from indicated cells using TRIzol reagent. DNA digestion was carried out after RNA extraction by deoxyribonuclease I. RNA quality was determined by examining A260/A280 with Nanodrop. RNA Integrity was confirmed by 1.5% agarose gel electrophoresis. Qualified RNAs were finally quantified by Qubit 3.0 with QubitTM RNA Broad Range Assay kit (Life Technologies, Q10210). Two micrograms of total RNAs were used for stranded RNA-seq library preparation using KCTM Stranded mRNA Library Prep Kit (Wuhan Seqhealth Co. Ltd. China, DR08402) for Illumina following the manufacturer’s instruction. PCR products corresponding to 200 to 500 bp were enriched, quantified, and finally sequenced on Novaseq 6000 sequencer (Illumina) with PE150 model. The RNA-seq data have been deposited in the GSA database under the accession number (HRA006231).

### Preparation of bone marrow–derived dendritic cells and bone marrow–derived macrophages

Bone marrow cells were isolated from C57BL/6J mouse tibia and femur and then cultured for 7 to 9 days in 10% FBS DMEM containing mouse granulocyte-macrophage colony-stimulating factor (GM-CSF, 50 ng/ml, Peprotech) for bone marrow–derived dendritic cells or M-CSF (50 ng/ml, Peprotech) for bone marrow–derived macrophages. IFN-β, TNF-α, and IL-1β were from MCE (MedChemExpress) and used at a final concentration of 20 ng/ml.

### Constructions

NSUN2 was constructed into the pCAGGS and pGEX6P-1 vector, respectively. SARS-CoV-2 N gene was constructed into the pCAG and pLVX vector, respectively. The other SARS-CoV-2 genes were constructed into the pCAG vector and pCDNA3.1 vector, respectively. The full-length genome of SARS-CoV-2 was divided into seven fragments and inserted respectively into a pUC57 vector. The gRNAs of NSUN2 were constructed into the lentiCRISPR-v2 vector, respectively.

### ER-DMVs fractionation

The ER-DMVs purification method was based on a previous article (*54*) and performed using the Endoplasmic Reticulum Isolation Kit (Sigma-Aldrich, ER0100) according to the manufacturer’s instructions.

### Construction of the trVLP

The construction method is based on the articles (*55*, *56*). In brief, the full-length genome of SARS-CoV-2 was divided into seven fragments and inserted respectively into a pUC57 vector, with a replacement of SARS-CoV-2 N gene by GFP. The seven fragments were amplified using Phanta Super-Fidelity DNA Polymerase (Vazyme, P501-d1). The obtained PCR product was digested by type IIS restriction endonuclease BsaI or BsmBI and then ligated with T4 ligase (NEB) to obtain a full-length cDNA template. The full-length template and N gene were in vitro transcribed and purified, respectively. Then, 20 μg of full-length mRNA and 10 μg of N mRNA were both added into a 4-mm cuvette (Bio-Rad) containing 0.4 ml of Caco-2–N cells (8 × 10^6^) in Ingenio Electroporation Solution (Mirus). A single electrical pulse was applied using a GenePulser apparatus (Bio-Rad) set at 270 V, 950 μF. The sample was placed at room temperature for 5 min and then cultured in a 10-cm cell culture dish for 3 days. Then, the supernatant was collected. This trVLP expresses a reporter gene (GFP) replacing SARS-CoV-2 N gene (SARS-CoV-2 GFP/ΔN trVLP). The complete viral life cycle can be achieved and confined in the cells ectopically expressing SARS-CoV-2 N proteins.

### Nuclear and cytoplasmic protein extraction

Nuclear and cytoplasmic extracts were obtained using Nuclear and Cytoplasmic Protein Extraction Kit (Beyotime, P0027) according to the manufacturer’s instructions.

### Ethics statement

This study was approved by the Ethics Committee of the Renmin Hospital of Wuhan University. The analyses of BALF samples and blood samples were performed on existing samples collected during standard diagnostic tests, posing no extra burden to patients. The sample collection was applied by Renmin Hospital of Wuhan University with written consent under appropriate institutional review board approval and was deidentified.
