# Supplementary material for: Epitranscriptomic m5C methylation of SARS-CoV-2 RNA regulates viral replication and the virulence of progeny viruses in the new infection
Source: Sci Adv. 2024 Aug 7;10(32):eadn9519. doi: 10.1126/sciadv.adn9519 (PMC11305390; doi:10.1126/sciadv.adn9519)
Supplement: Supplementary file 1 — Figs. S1 to S13 Tables S1 and S2 [file sciadv.adn9519_sm.pdf]

Science Advances
AAAS

Supplementary Materials for

Epitranscriptomic m^5C methylation of SARS-CoV-2 RNA regulates viral replication and the virulence of progeny viruses in the new infection

Hongyun Wang *et al.*

Corresponding author: Yu Chen, chenyu@whu.edu.cn

Sci. Adv. **10**, eadn9519 (2024)
DOI: 10.1126/sciadv.adn9519

This PDF file includes:

Figs. S1 to S13
Tables S1 and S2

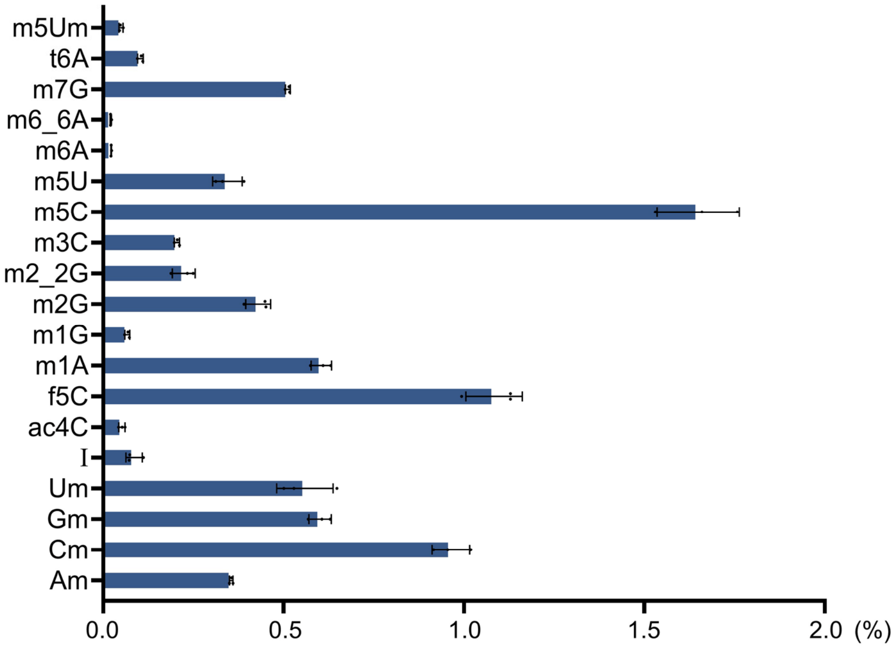

fig. S1. RNA modifications in SARS-CoV-2 GFP/ΔN trVLP by LC-MS/MS. SARS-CoV-2 GFP/ΔN trVLP was purified and subjected to LC-MS/MS to detect the RNA modifications.

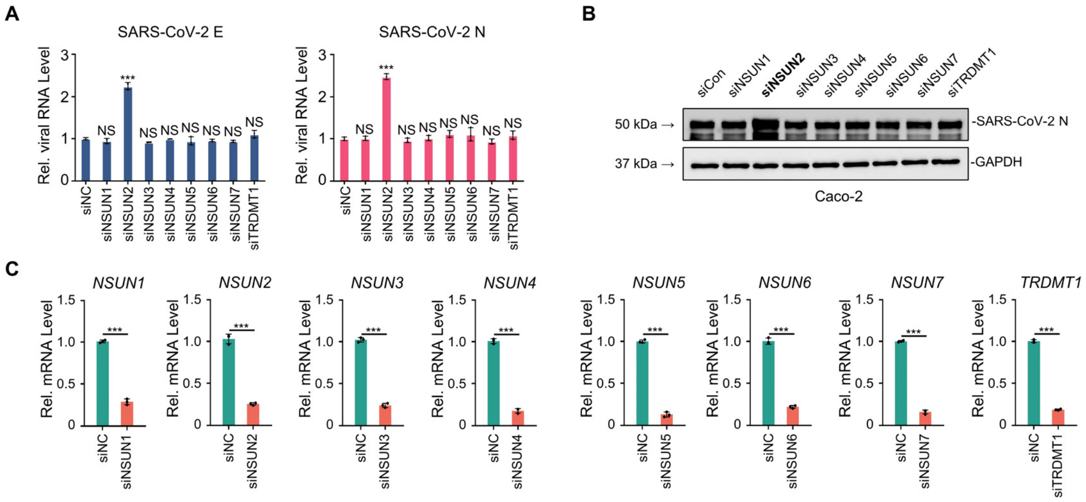

fig. S2. The screening tests of NOP2/SUN RNA methyltransferase family in regulation of SARS-CoV-2 replication. (A) qPCR analysis of RNA levels of N or E in Caco-2 cells transfected with siControl or siRNAs for 36 h, with infection by SARS-CoV-2 at an MOI = 0.02 for another 24 h. (B) Western blot analysis of protein levels of N in Caco-2 cells transfected with siControl or siRNAs for 36 h, with infection by SARS-CoV-2 at an MOI = 0.02 for another 24 h. (C) qPCR analysis of mRNA levels of the indicated genes in Caco-2 cells.

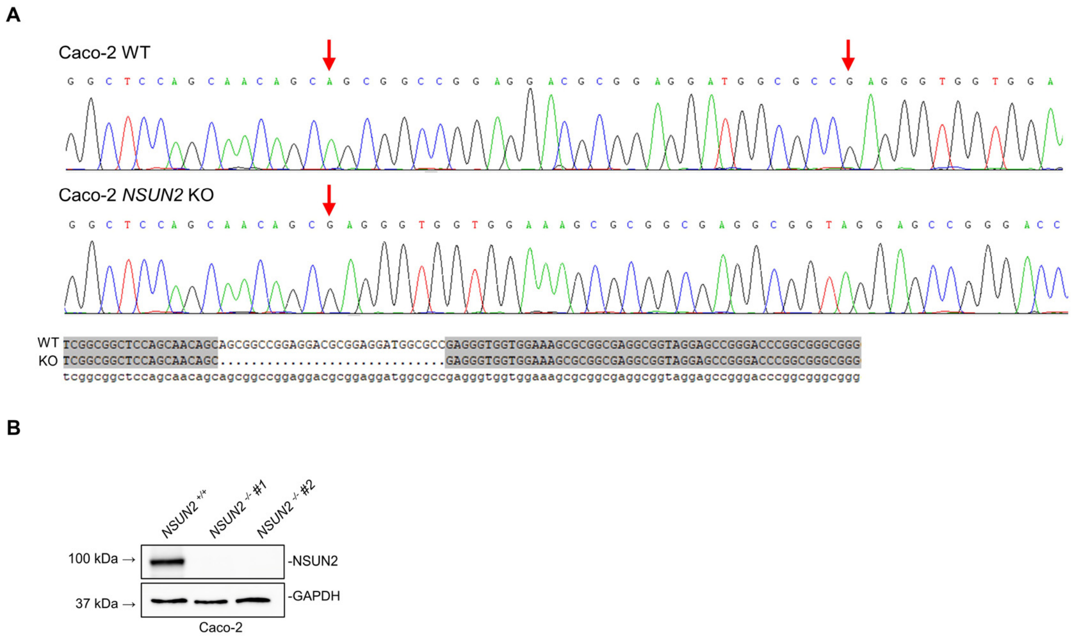

fig. S3. Construction of NSUN2 knockout cell line in Caco-2 cell. (A) Sequencing results of Caco-2 WT and NSUN2 KO single cell line. (B) Western blot results of Caco-2 WT and NSUN2 KO single cell line.

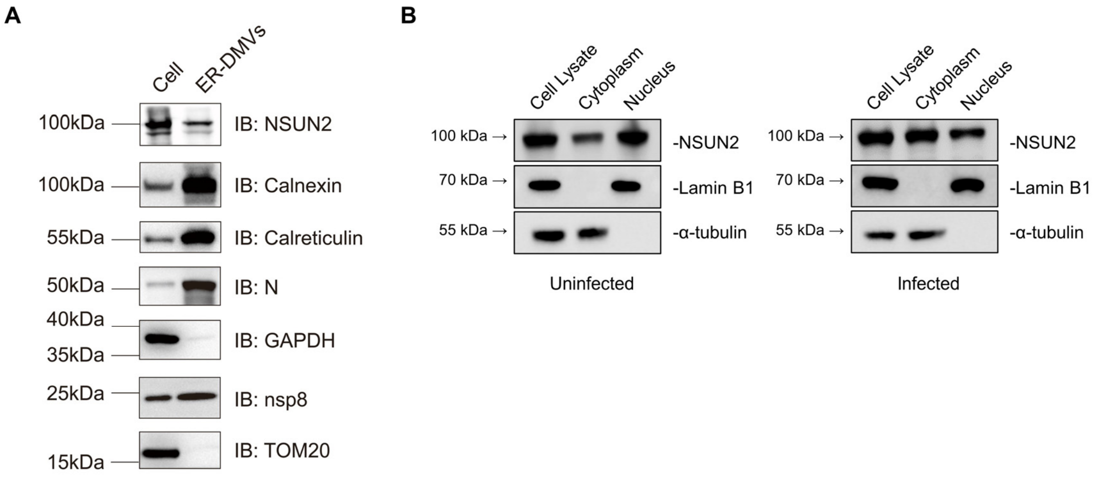

fig. S4. The subcellular distribution of endogenous NSUN2 upon SARS-CoV-2 infection. (A) The identification of proteins in purified ER-DMVs from SARS-CoV-2 GFP/ΔN trVLP infected Caco-2-N cells. Calnexin and Calreticulin proteins are localized in ER as controls. TOM20 protein is localized in mitochondria as a control. (B) Nuclear and cytoplasmic protein extraction of uninfected or from SARS-CoV-2 GFP/ΔN trVLP infected Caco-2-N cells to investigate the subcellular localization of endogenous NSUN2 upon SARS-CoV-2 infection.

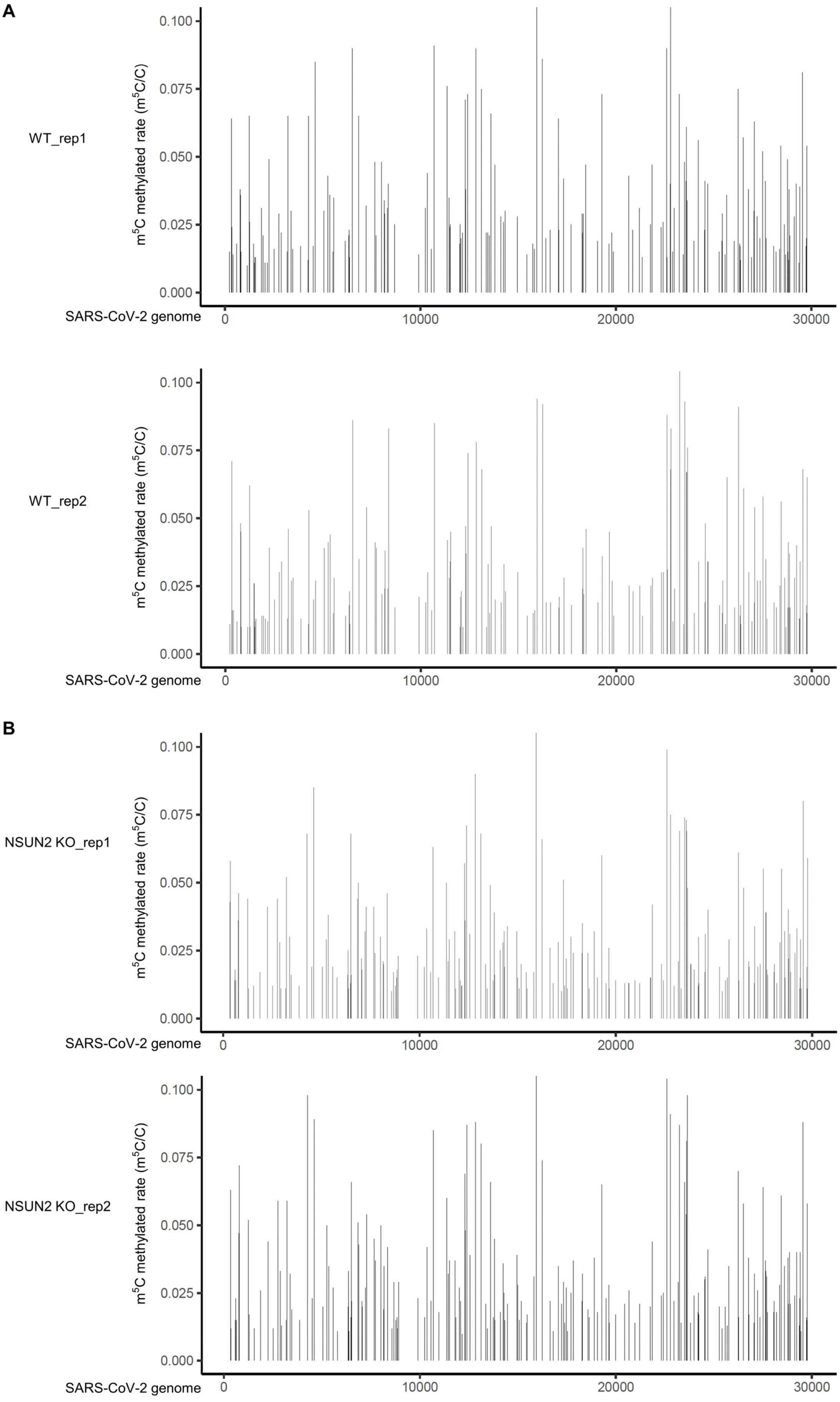
A
WT_rep1
WT_rep2
B
NSUN2 KO_rep1
NSUN2 KO_rep2
m5C methylated rate (m5C/C)
SARS-CoV-2 genome
0.000
0.025
0.050
0.075
0.100
0
10000
20000
30000

fig. S5. Bis-seq results of m^5C methylated sites along SARS-CoV-2 positive-sense RNA. The identified m^5C methylated sites along SARS-CoV-2 positive-sense RNA in SARS-CoV-2-infected wild-type Caco-2 cells (A) and NSUN2 knockout Caco-2 cells (B), related to Fig. 2I.

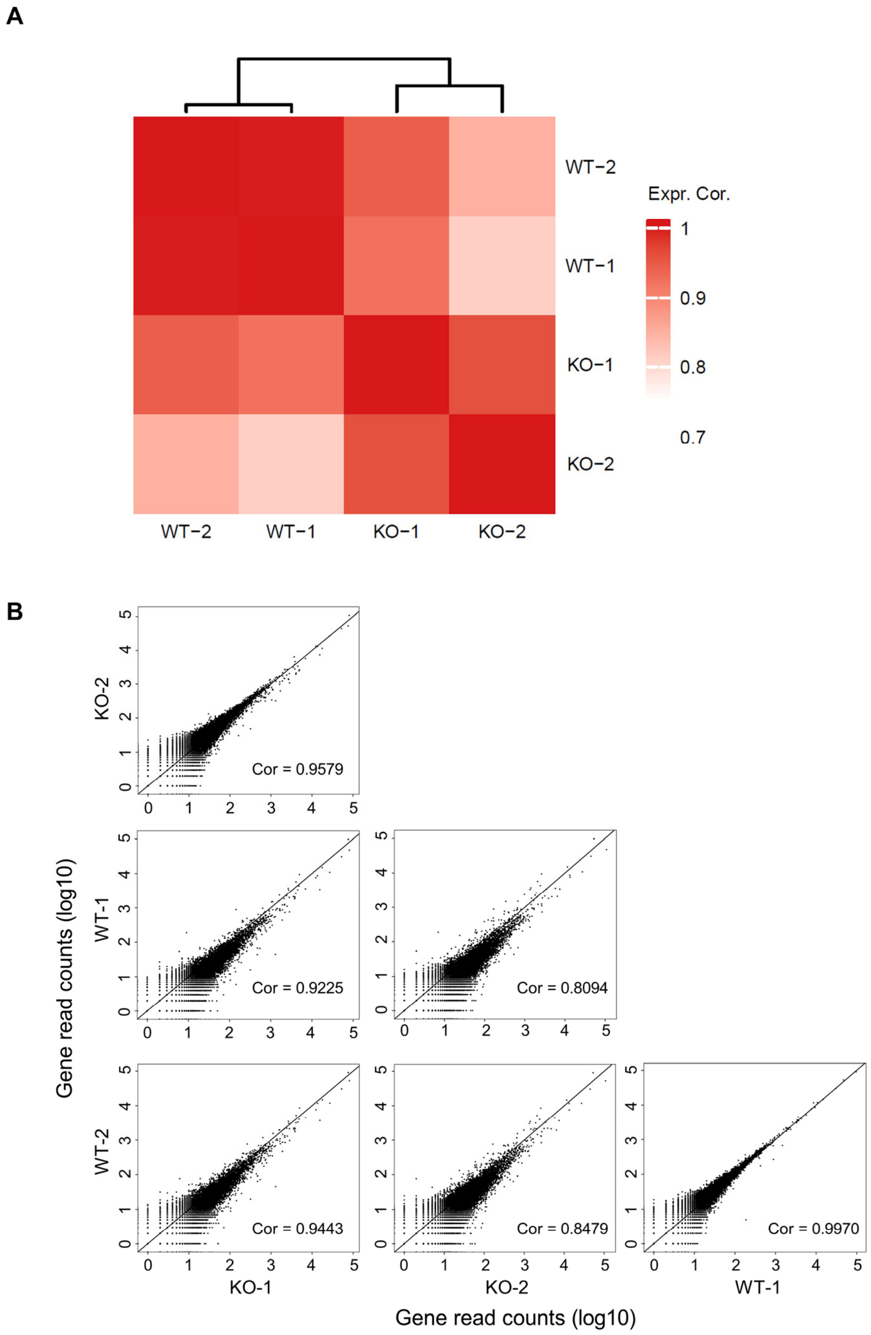

fig. S6. Correlation and clustering analysis of RNA Bis-seq, related to fig. S5. Based on the Person correlation coefficient, the RPKM value of RNA Bis-seq is used to calculate the correlation and draw the graph (A-B).

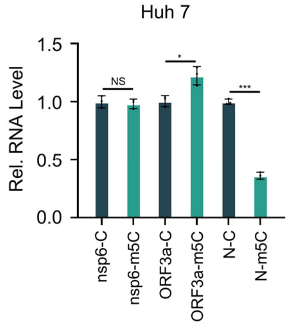

fig. S7. The different function of incorporation of m^5C in nsp6, ORF3a and N. qPCR analysis of RNA levels of corresponding gene in Huh7 cells transfected with transcribed RNAs with C or m^5C for 10 h.

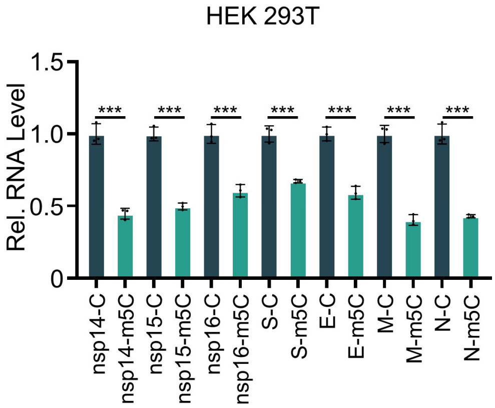

fig. S8. The incorporation of m^{5}C in several SARS-CoV-2 RNA segments resulted in RNA degradation in HEK293T cells. qPCR analysis of RNA levels of corresponding gene in HEK293T cells transfected with transcribed RNAs with C or m^{5}C for 10 h.

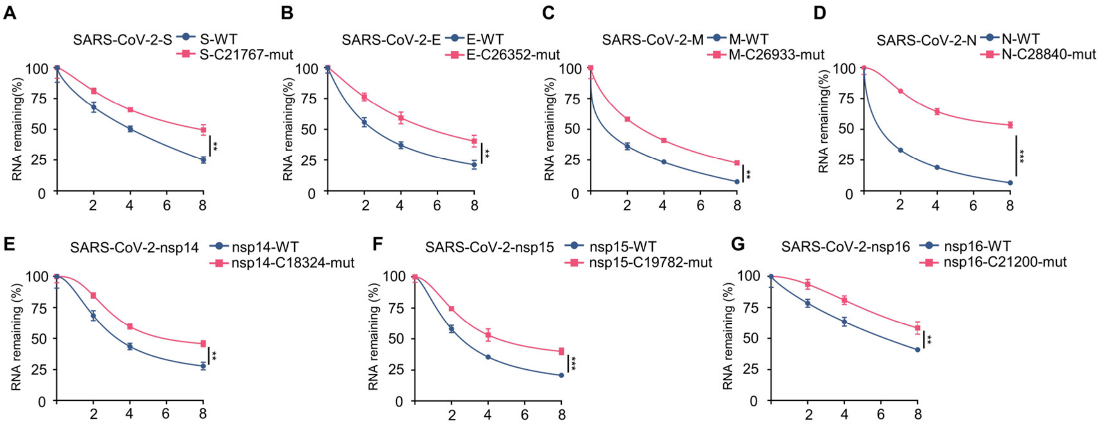

fig. S9. The screened m^{5}C methylated cytosines in SARS-CoV-2 were identified to regulate RNA degradation in HEK293T cells. (A-G) Stability analysis of RNA of each gene in HEK293T cells transfected with wild-type (WT) or m^{5}C modification site-mutations (mut) of each gene for 24 h, with treatment of actinomycin D (ActD) for another 0, 2, 4, and 8 h.

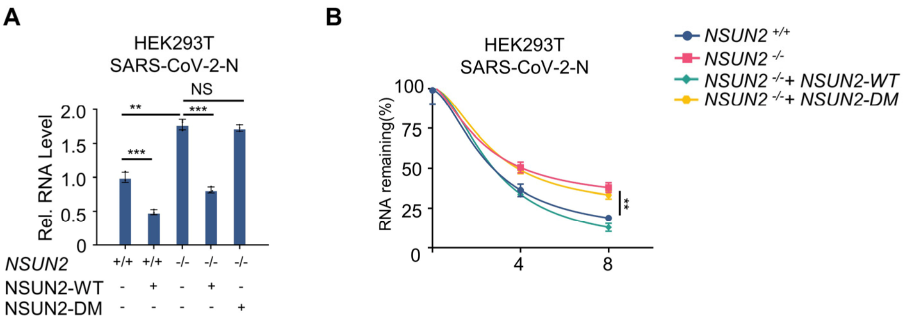

fig. S10. NSUN2 regulated RNA level of SARS-CoV-2 N gene depending on its methyltransferase activity in HEK293T cells. (A) qPCR analysis of RNA levels of N gene in wild-type HEK293T cells (*NSUN2*$^{+/+}$) or NSUN2 knockout cells (*NSUN2*$^{-/-}$), transfected with SARS-CoV-2 N and wild-type NSUN2 (NSUN2-WT) or I302A/C321A double mutation (NSUN2-DM). (B) Stability analysis of RNA levels of N gene in wild-type HEK293T cells (*NSUN2*$^{+/+}$) or NSUN2 knockout cells (*NSUN2*$^{-/-}$), transfected with SARS-CoV-2 N and wild-type NSUN2 (NSUN2-WT) or I302A/C321A double mutation (NSUN2-DM) for 24 h, with treatment of actinomycin D (ActD) for another 0, 4, and 8 h.

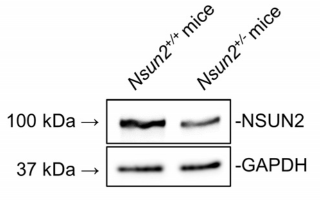

fig. S11. The NSUN2 expression levels in *Nsun2*$^{+/+}$ mice and *Nsun2*$^{+/-}$ mice. Western blot results of NSUN2 protein in the lung tissues of *Nsun*$^{+/+}$ mice or *Nsun*$^{+/-}$ mice.

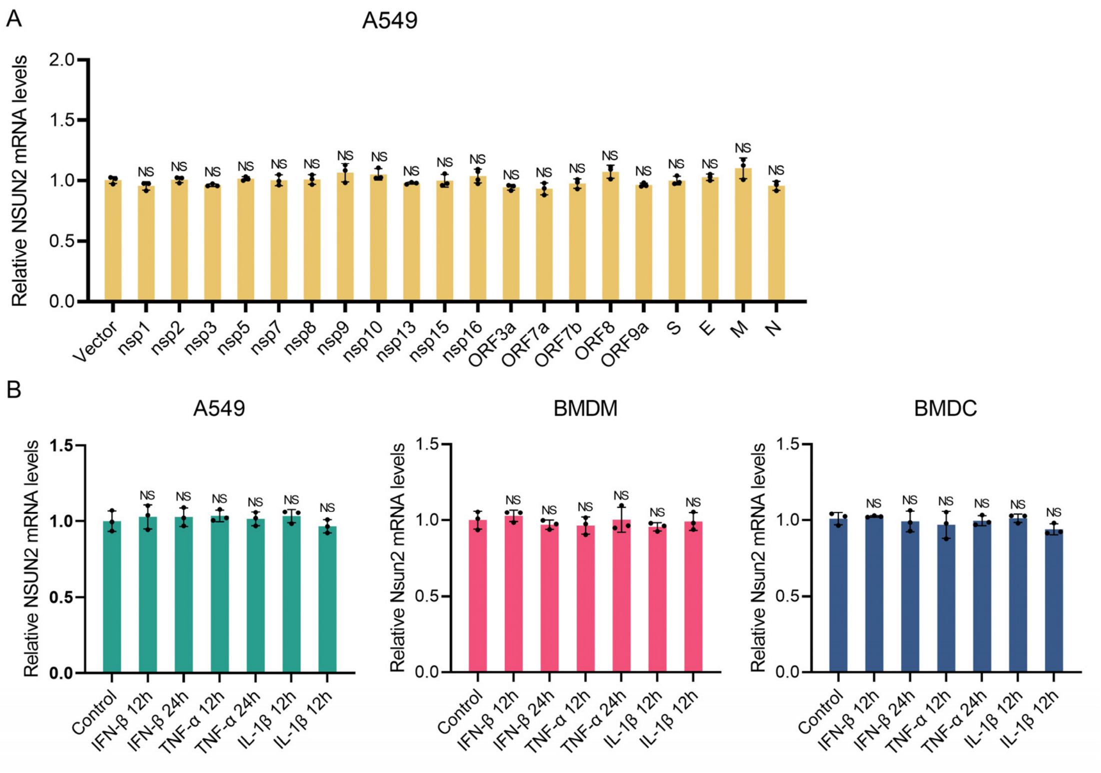

fig. S12. SARS-CoV-2 proteins or treatment of IFN-β, TNF-α and IL-1β didn't affect NSUN2 mRNA levels. (A) qPCR analysis of mRNA levels of NSUN2 in A549 cells transfected with different SARS-CoV-2 proteins for 48 h. (B) qPCR analysis of mRNA levels of NSUN2 in A549 cells, BMDMs or BMDCs, with treatment of IFN-β, TNF-α and IL-1β for different time as indicated.

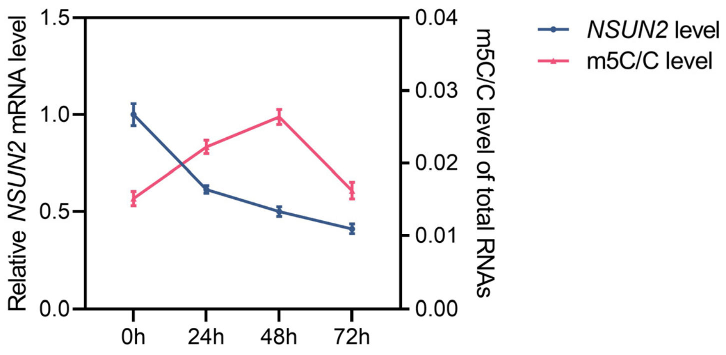

fig. S13. The kinetics of m^{5}C modification mediated by NSUN2 and NSUN2 expression levels during SARS-CoV-2 infection. qPCR analysis of mRNA levels of NSUN2 in Caco-2 cells infected with SARS-CoV-2 for different time points. Analysis of m^{5}C/C ratio using LC-MS/MS of total RNAs extracted from SARS-CoV-2-infected Caco-2 cells infected with SARS-CoV-2 for different time points. MOI = 0.02.

table S1. Primers for mRNA Quantification

	Forward	Reverse	Probe (If necessary)
SARS-CoV-2 S	GCACACGCCTATTAATTTAGTGC	CACCAGCTGTCCAACCTGAAG	
SARS-CoV-2 M	ATCACCGGTGGAATTGCTATC	CTGGTCAGAATAGTGCCATGG	
SARS-CoV-2 E	ACAGGTACGTTAATAGTTAATAGCGT	ATATTGCAGCAGTACGCACACA	ACACTAGCCATCCTTACTGCGCTTCG (Probe-5`6-FAM, 3`BHQ1)
SARS-CoV-2 N	TAATCAGACAAGGAACTGATTA	CGAAGGTGTGACTTCCATG	GCAAATTGTGCAATTTGCGG (Probe-5'HEX,3'BHQ-1)
GAPDH	CAGCCTCAAGATCATCAGCA	TGTGGTCATGAGTCCTTCCA	CTGCTTAGCACCCCTGGCCA(Probe-5`CY5,3`BHQ3)
mGapdh	TGCACCACCAACTGCTTAG	GGATGCAGGGATGATGTTC	CAGAAGACTGTGGATGGCCCCTC(Probe-5`CY5,3`BHQ3)
SARS-CoV-2 nsp14	TTGTTAAGCGTGTTGACTG	CCAATGTCGTGAAGAACTG	
SARS-CoV-2 nsp15	GGACATTGCTGCTAATACTG	GACCATCAACTCTACCATCA	
SARS-CoV-2 nsp16	GGTGTTGCTATGCCTAATCT	TGCGACATTCATCATTATGC	
SARS-CoV-2 ORF3a	CACAGTTACTTCACTTCAGAC	TCTTCAGGCTCATCAACAAT	
SARS-CoV-2 nsp6	GGTCTATATGCCTGCTAGTTGGG	CGAGTGTCAAGACATTCATAAGTG	
NSUN2	CAAGCTGTTCGAGCACTACTAC	CTCCCTGAGAGCGTCCATGA	
TRDMT1	CGGGTGCTGGAGCTATACAG	CGACAGTGTTGACATCAATGGC	
METTL3	TTGTCTCCAACCTTCCGTAGT	CCAGATCAGAGAGGTGGTGTAG	
METTL14	AGTGCCGACAGCATTGGTG	GGAGCAGAGGTATCATAGGAAGC	
mNsun2	AGGTGGCTATCCCGAGATCG	GACTCCATGAATTGGTCCCATT	
NSUN1	AAGGGTGCCGAGACAGAACT	GAGCACGACTAGACAGCCTC	
NSUN3	CATGCTGGCAATATGCTGTCC	AAAGATCCCTGAGAGAGTGTGT	
NSUN4	CCATCAATCCGTGTCAGTCTC	GCTTAGCACTTACATGATCCCAG	
NSUN5	CGCTACCATGAGGTCCACTAC	GCATCTCGCACCACGTCTT	
NSUN6	TTAAGAGGAGCCCATGTCTATGC	CTTTGCGGCTTAGTTCAGAAATC	
NSUN7	GGACTCCGTTTATGTCATGGC	CTCAGACTCGGACAAGGACC	

table S2. Mutations of the identified m5C sites				
seqContext	Location	Position	Mutation	Mutation type
actagagaagCtgttggtacc	nsp14	18324	GCT-GAT	missense mutation
taaaccaccgCCtggagatca	nsp14	18449&18450	GCC-GAA	missense mutation
gagctttgggCtaagcgcaac	nsp15	19782	GCT-GAT	missense mutation
acaatctagtCaagcgtggca	nsp16	20651	TCA-TAA	missense mutation
cattttggtgCtggttctgat	nsp16	20859	GCT-GAT	missense mutation
gctcatgggaCacttcgcatg	nsp16	21200	ACA-AAA	missense mutation
tctctgggacCaatggtacta	S	21767	CCA-CAA	silent mutation
ttataattacCtgtatagatt	S	22902	CCT-CTT	silent mutation
caacttactcCtacttggcgt	S	23440	CCT-CAT	missense mutation
atccttactgCgcttcgattg	E	26337	GCG-GAG	missense mutation
cgattgtgtgCgtactgctgc	E	26352	GCG-GAG	missense mutation
taacgtgagtCttgtaaaacc	E	26381	CTT-TTA	silent mutation
gtaatcggagCtgtgatcctt	M	26933	GCT-GAT	missense mutation
actgagggagCcttgaataca	N	28672	GCC-GAC	missense mutation
cgcaacagttCaagaaattca	N	28840	TTC-TTT	silent mutation
